# Leveraging genomics to understand threats to migratory birds

**DOI:** 10.1111/eva.13231

**Published:** 2021-04-10

**Authors:** Brenda Larison, Alec R. Lindsay, Christen Bossu, Michael D. Sorenson, Joseph D. Kaplan, David C. Evers, James Paruk, Jeffrey M. DaCosta, Thomas B. Smith, Kristen Ruegg

**Affiliations:** ^1^ Department of Ecology and Evolutionary Biology University of California Los Angeles California USA; ^2^ Center for Tropical Research Institute of the Environment and Sustainability University of California Los Angeles California USA; ^3^ Department of Biology Northern Michigan University Marquette Michigan USA; ^4^ Department of Biology Colorado State University Fort Collins Colorado USA; ^5^ Department of Biology Boston University Boston Massachusetts USA; ^6^ Common Coast Research and Conservation Hancock Michigan USA; ^7^ Biodiversity Research Institute Portland Maine USA; ^8^ Biology Department Saint Joseph’s College Standish Maine USA; ^9^ Biology Department Boston College Chestnut Hill Massachusetts USA

**Keywords:** botulism, Common Loon, conservation genomics, disease, *Gavia immer*, RAD sequencing, waterbirds, wildlife management

## Abstract

Understanding how risk factors affect populations across their annual cycle is a major challenge for conserving migratory birds. For example, disease outbreaks may happen on the breeding grounds, the wintering grounds, or during migration and are expected to accelerate under climate change. The ability to identify the geographic origins of impacted individuals, especially outside of breeding areas, might make it possible to predict demographic trends and inform conservation decision‐making. However, such an effort is made more challenging by the degraded state of carcasses and resulting low quality of DNA available. Here, we describe a rapid and low‐cost approach for identifying the origins of birds sampled across their annual cycle that is robust even when DNA quality is poor. We illustrate the approach in the common loon (*Gavia immer*), an iconic migratory aquatic bird that is under increasing threat on both its breeding and wintering areas. Using 300 samples collected from across the breeding range, we develop a panel of 158 single‐nucleotide polymorphisms (SNP) loci with divergent allele frequencies across six genetic subpopulations. We use this SNP panel to identify the breeding grounds for 142 live nonbreeding individuals and carcasses. For example, genetic assignment of loons sampled during botulism outbreaks in parts of the Great Lakes provides evidence for the significant role the lakes play as migratory stopover areas for loons that breed across wide swaths of Canada, and highlights the vulnerability of a large segment of the breeding population to botulism outbreaks that are occurring in the Great Lakes with increasing frequency. Our results illustrate that the use of SNP panels to identify breeding origins of carcasses collected during the nonbreeding season can improve our understanding of the population‐specific impacts of mortality from disease and anthropogenic stressors, ultimately allowing more effective management.

## 1 INTRODUCTION

Migratory bird species are in decline (Robbins et al., 1989; Rosenberg et al., 2019; Sanderson et al., 2006). Most efforts to understand the causes of these declines have focused on reproductive success on the breeding grounds (Both et al., 2009; Burke & Nol, 2000; Robinson et al., 1995). While events during the reproductive part of the annual cycle can have significant impacts on the ecology and demography of populations, they comprise only a part of the annual cycle of most species (Marra et al., 2015). For most migrant species, we know comparatively little about the nonbreeding period of their life cycle. Migrating and overwintering birds may travel vast distances during the course of which they may encounter multiple natural and anthropogenic threats including adverse weather, disease, habitat loss, and the danger of striking human‐made structures such as buildings, cell towers, or wind turbines (Erickson et al., 2014; Longcore et al., 2012; Loss et al., 2014). Even if a bird survives these threats, stressors experienced during migration and overwintering can negatively impact their reproductive success in the following breeding season (Inger et al., 2010). For example, several studies have found that habitat quality on the wintering area influences reproductive success on the breeding grounds (Marra et al., 1998; Norris et al., 2004; Saino et al., 2004). Such inter‐seasonal effects may also happen in the reverse; for example, mercury contamination in the breeding range has been shown to reduce over‐winter survival in long‐distance migrants (Ma et al., 2018).

Among the many threats faced by migrant birds are the diseases they may encounter across the annual cycle (Altizer et al., 2011). How migration impacts disease threats is a complex issue given that it can increase cross‐species transmission (Figuerola & Green, 2000; Krauss et al., 2010; Waldenström et al., 2002), but can also lower the risk of spread within species (Altizer et al., 2011; Bartel et al., 2011; Bradley & Altizer, 2005). Making matters more complex, migration routes and timing are shifting due to climate change and other anthropogenic disturbances (Robinson et al., 2009; Saino et al., 2010; Wilcove & Wikelski, 2008), which has the potential to alter transmission dynamics (Hall et al., 2016; Mysterud et al., 2016; Satterfield et al., 2016).

Given that migratory birds spend the majority of their lives outside of their breeding grounds, it is essential to understand the impacts of the entire life cycle on population demography. Further, an understanding of the degree of connectivity among populations can help predict how widespread or targeted the impacts of environmental stressors in a wintering area are likely to be on breeding populations and vice versa. Understanding migratory movements and connectivity will also be essential for identifying disease transmission routes (Altizer et al., 2011; Fuller et al., 2012). Thus, understanding connectivity is both generally important for conservation (Altizer et al., 2011; Hall et al., 2016) and specifically relevant to epidemiological analyses (Fritzsche McKay & Hoye, 2016; Rappole et al., 2000; Webster et al., 2002). Techniques and technologies such as bird banding, satellite tracking, and radar have added significantly to our understanding of avian migration and continue to do so (Beingolea & Arcilla, 2020; Berthold et al., 1992; Delmore et al., 2012; Egevang et al., 2010; Gill et al., 2009; Wikelski et al., 2007). These tracking approaches can help link wintering and breeding grounds and can provide detailed information about routes, timing, and even environmental and physiological conditions (Bridge et al., 2011). However, many species still cannot be tracked by these methods because power requirements render the transmitters too cumbersome for most species, and the number of individuals that can be studied is often limited by high costs, the need for nearby receivers, or the need to recapture the bird to retrieve the data (Bridge et al., 2011). Some of these limitations may soon change (Wikelski et al., 2007), but currently, a comprehensive understanding remains limited by a lack of information on the linkages and routes between wintering and breeding grounds for most species (Bowlin et al., 2010; Faaborg et al., 2010). Genomic approaches to studying migration can provide an alternative, or even complementary approach, in that large numbers of individuals can be studied at relatively low cost.

**FIGURE 1:**
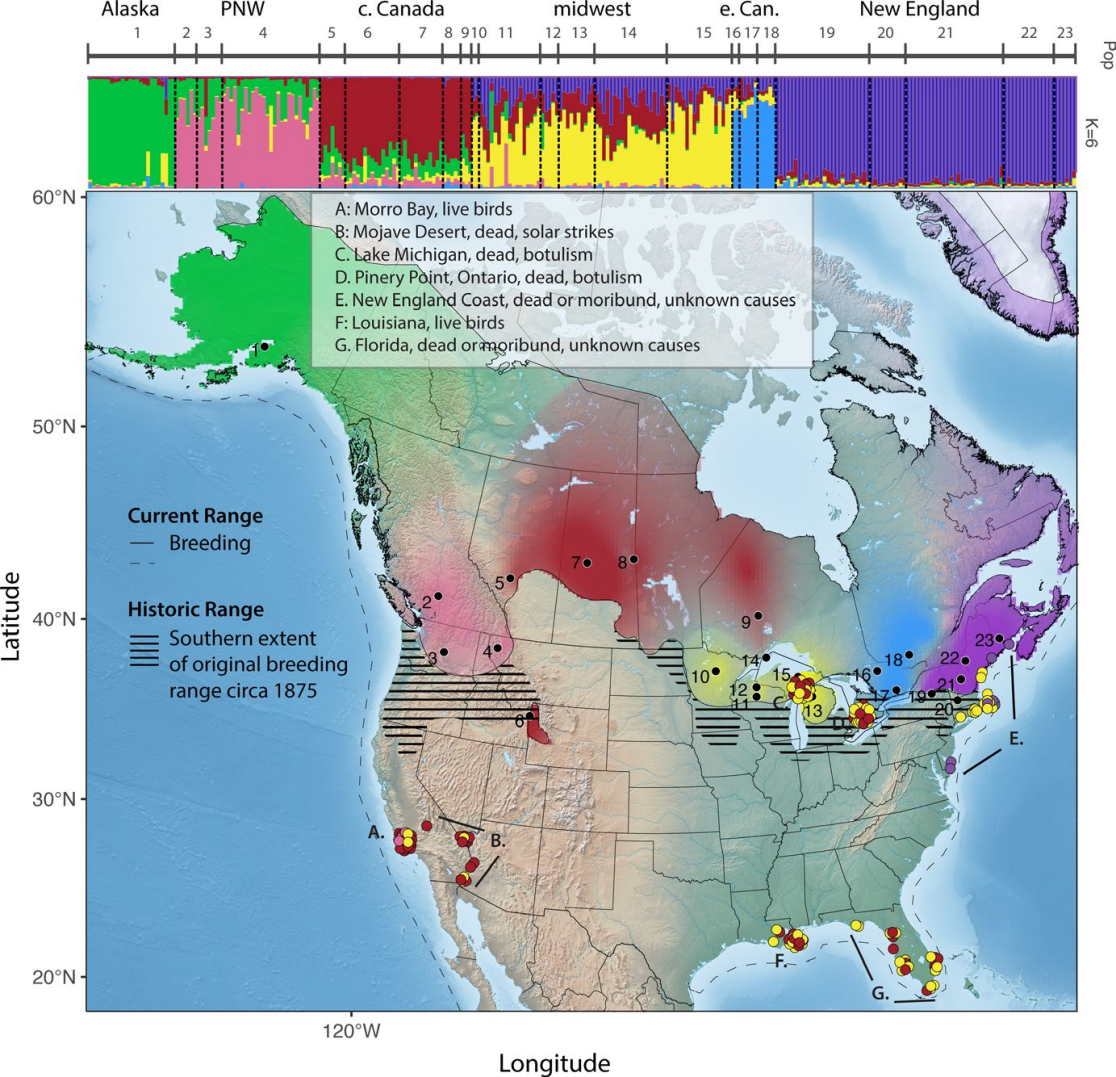
Conservation units and assignments of migrating and wintering common loon identified using SNP‐based genetic markers (Fluidigm). The numbers in both panels correspond to the locations listed in Table 1. Top panel: six genetically differentiated conservation units across the breeding grounds based on *STRUCTURE* analysis. Alaska (green), Pacific Northwest (pink), central Canada (red), Midwest (yellow), eastern Canada (blue), and New England (purple). Bottom panel: spatially explicit population structure across the annual cycle. The colors across the breeding range represent the ancestry results from the *STRUCTURE* analysis, which were postprocessed using R so that the density of each color reflects the relative posterior probability of membership for each pixel to the most probable of the six different clusters (see text). The results were clipped to the species distribution map (NatureServe, 2012). Color‐filled data points indicate migratory or wintering birds and their assignment to one of the breeding conservation units. 142 of 164 birds were assignable to a conservation unit. Zoomed‐in maps showing the locations of the assigned samples can be found in Figure S6

The common loon (*Gavia immer*, *Brünnich 1764*) is an iconic migratory waterbird species of North America whose populations have been generally stable because the bulk of the population breeds in Canadian lakes, far from the anthropogenic habitat degradation seen on the southern fringe of their range (Evers et al., 2020). Nonetheless, as a long‐lived species with low fecundity their conservation status is listed as “vulnerable” and breeding populations have retracted from the southern part of their breeding range (Figure 1), in some cases disappearing from former breeding sites (Evers, 2007). As a result, they are listed as a species of moderate concern by the North American Waterbird Conservation Plan (Kushlan et al., 2002) and as threatened or “of concern” in nine states in the United States (Paruk et al., 2014). In addition to their status as a species of concern, loons are an important indicator species due to their need for clear, unpolluted lakes and their sensitivity to a variety of anthropogenic threats (Evers, 2006). Specific issues faced by loons on breeding lakes include loss and degradation of habitat, climate change factors related to increased cyanobacteria toxin blooms, contaminants such as lead and mercury, water level management on dammed waterbodies, and disease (Burgess & Meyer, 2008; Evers et al., 2011, 2020; Mitro et al., 2008; Scheuhammer et al., 2007; Warden, 2010), some of which may have synergistic effects (Paerl & Paul, 2012).

In particular, common loons breeding in some areas of Canada have experienced significant declines in productivity over the last four decades, largely due to mercury and the compounding effects of acid precipitation (Bianchini et al., 2020; Tozer et al., 2013). Further, populations are exposed to stressors during the migratory period, including from oil spills (Evers et al., 2019), collisions with cell towers, buildings, and solar/wind installations (Goodale & Milman, 2014), as well as increased risk of exposure to botulism, a lethal paralytic disease caused by a neurotoxin produced by the bacterium *Clostridium botulinum* (Anza et al., 2016). Outbreaks of botulism type E occur episodically in the Great Lakes region, a critical staging area and breeding ground for common loons (Evers, 2007; Kenow et al., 2018), and climate change is expected to exacerbate the frequency and intensity of botulism outbreaks (Lafrancois et al., 2011; Mooij et al., 2005). As a result of the multiple stressors that common loons are exposed to across their annual cycle, information on population genetic structure and connectivity can be used to predict the consequences of anthropogenic threats and disease outbreaks and to inform proposed reintroductions.

Here, we developed a genome‐wide panel of single‐nucleotide polymorphisms (SNPs) for common loons using samples collected from across the breeding range and used it to create a map of genetic variation across geographic space, or *genoscape* (Ruegg et al., 2014) for the species which can serve as a foundation for assessing population stability and impacts of threats outside the breeding grounds. Specifically, our goals are to: (1) make a preliminary assessment of population structure in common loons, (2) create a genoscape that delineates genetically differentiated subpopulations which we designate and henceforth refer to as conservation units (i.e., populations for assignment purposes as defined in (Coates et al., 2018; Funk et al., 2012), (3) assign migrating and wintering loons back to their conservation units of origin in order to assess migratory connectivity, and (4) discuss current and future threats in the context of the patterns found. Current results and future application of this approach are expected to improve conservation decision‐making by better informing managers about population impacts across the annual cycle.

## 2 METHODS

### 2.1 Sample collection

Through a large collaborative effort, we collected samples from breeding, wintering, and migrating common loons from across North America during the years 1979 and 1992–2016. Samples for genetic analysis included blood samples collected from birds captured in known breeding areas across North America during the breeding season, and samples collected from wintering or migratory birds, including the following: (1) feathers from bird carcasses resulting from botulism outbreaks in the Great Lakes or collisions at solar facilities in southern California, (2) blood or feathers collected from birds that were moribund or dead from mostly unknown causes from Florida and the northeastern seaboard, and (3) blood from living, healthy birds from Morro Bay, California, and coastal Louisiana. For loons that were sampled on the Great Lakes, which serve primarily as stopover sites, we used both collection date and location on the lakes to assess whether birds were breeding or migrating (breeding birds are more likely to be found on or near the few islands where they breed, whereas migrating birds are more likely to be found on mainland beaches). Tissue samples and previously extracted DNA were sent to UCLA for library preparation and/or SNP genotyping. Samples were transferred to UCLA under a protocol approved by the Research Safety and Animal Welfare Administration, University of California Los Angeles: ARC# 2017‐073‐03, approved 10/28/2017.

### 2.2 DNA isolation

Qiagen's DNeasy 96 blood and tissue kit (Qiagen Inc.) was used to extract DNA from each sample. The amount of starting material varied by sample type: we used between 10 and 50 µl of blood for whole blood, 10 mg of sample for tissues and at least one calamus for feathers. Each sample was incubated overnight at 56°C in 20 µl Proteinase K and 180 µl of lysis buffer. To break down the keratin in feathers, 10 µl of Dithiothreitol (DTT) was also added to the lysis mix. All samples were eluted the next day per the manufacturer's protocol, yielding a final elution of 120 µl of DNA solution per feather sample and 200 µl per blood or tissue sample.

### 2.3 ddRAD sequencing and SNP discovery

In a preliminary effort to characterize population structure, we employed a double‐digest restriction site‐associated (ddRAD) sequencing method detailed in (DaCosta & Sorenson, 2014) and assessed its utility in detecting fine scale population structure. We used this dataset to run a principal components analyses (PCA). Further details of the ddRAD methods can be found in the supplement. Preliminary analysis of the ddRAD dataset suggested that it did not have sufficient power to assign individuals to populations. However, as this was the only dataset containing sufficient samples from Alaska, we retained these data to help inform the design of our genotyping assay.

### 2.4 RAD‐PE sequencing and SNP discovery

To identify SNPs useful for delineating breeding populations of the common loon across North America, we augmented our sampling with an additional 204 individuals selected from across the North American breeding range and used restriction site‐associated DNA paired‐end (RAD‐PE) sequencing to generate additional markers. RAD‐PE libraries were constructed at UCLA following Ruegg et al. (2014) using the restriction enzyme SbfI. The individually barcoded libraries were sequenced on an Illumina HiSeq 2000 (Illumina) using paired‐end 150 base pair sequencing reads. Paired‐end sequences were de‐multiplexed and stripped of individual barcodes using Stacks 1.37 (Catchen et al., 2013), resulting in reads 140 bp in length. The resulting sequences were mapped to the red‐throated Loon (*Gavia stellata*) genome assembly (Zhang et al., 2014) using *Bowtie2* (Langmead & Salzberg, 2012), with the sensitive local option. Alignment rate was 86% (range 9.65–92.02). Only six (first reads) and five (second reads) samples had fewer than 75% of their reads align. Duplicate paired‐end reads for a given sample were removed using *Samtools* 0.1.19 (Li et al., 2009).

We called SNPs using the GATK 4.9.3 Haplotype Caller (McKenna et al., 2010). Genotypes were called for all positions with genotype quality of 20 or greater. In an initial round of filtering, we removed indels and kept SNPs according to the following parameters: minimum genotype quality = 30, read depth >= 8, biallelic and minimum allele frequency (MAF) >0.01. We completed a second round of filtering using *genoscapeRtools* (https://github.com/eriqande/genoscapeRtools; https://doi.org/10.5281/zenodo.848279), a R software program that visualizes the tradeoff between discarding SNPs with low coverage and discarding individuals with missing genotypes. Here, we discarded low quality SNPs and low coverage individuals for a final set of variants with <20% missing genotypes per SNP and <25% missing data per individual. This final set was used to assess population structure and develop a downstream SNP panel for population assignment.

### 2.5 Initial assessment of breeding population structure

Because there was little existing information about genetic population structure for common loons (Dhar et al., 1997; McMillan et al., 2004), we made an initial assessment of breeding population structure using both the ddRAD and RAD‐PE SNP datasets. The ddRAD data were coded following the approach of (Novembre & Stephens, 2008) and a PCA was run in R (R Core Team, 2020). Family structure and linkage can confound efforts to assess population structure (Anderson & Dunham, 2008; Chatfield & Collins, 1981; Conomos et al., 2015; Zou et al., 2010). Therefore prior to running a PCA using the RAD‐PE dataset, we assessed relatedness using King (Manichaikul et al., 2010), removing one individual of any pair related to each other to a degree greater than half sib or first cousin. Finally, we performed pairwise pruning in PLINK (Purcell et al., 2007) to remove one of any pair of SNPs with an *r* ^2^ greater than 0.2. In all, 129 individuals (Table S2) and 39,912 markers from the RAD‐PE dataset were used for the assessment of population structure by PCA using the program SNPrelate (Zheng et al., 2012).

For the 16 locations with at least four individuals remaining after filtering for missing data, we calculated pairwise *F*_ST_ across all quality filtered SNPs using the R package *assigner* version 0.5. 6 (Gosselin, 2020). Here, we used the *hierfstat* model (Goudet, 2005) to also provide confidence intervals surrounding the *F*_ST_ estimates. We also estimated pairwise *F*_ST_ between the five conservation units represented in the RAD‐PE dataset (the Alaska conservation unit did not have enough individuals after filtering). We assessed isolation by distance among 16 sampling regions, regressing pairwise *F*_ST_ values on the geographic distance between the centroids of each region using a Mantel test in the vegan package (Oksanen et al., 2018) in R, randomly permuting the geographic locations 999 times.

### 2.6 Development of genotyping assay

Starting with the final RAD‐PE dataset (see RAD‐PE sequencing and SNP discovery), we used custom R scripts to identify a set of 259 SNPs with the largest pairwise allele frequency differences among the conservation units identified by the analysis of population structure described above (Figures 1, and S1,S2). From this initial list of divergent variants, we created a low‐cost assay to screen additional individuals from across the range. This approach allowed us to assay SNP genotypes at a fraction of the cost (1/4 or less) of using RAD‐seq for every sample, making it a more cost‐effective strategy for processing the hundreds of samples used in this study. We designed the assay such that the number of SNPs used to delineate each pair of conservation units was inversely proportional to the degree of divergence between them. We used the R package SNPS2ASSAYS (Anderson, 2015) to evaluate which of our top‐ranking SNPs would generate designable assays for each conservation unit. Assays were considered designable if GC content was less than 0.65 over the 200 bp surrounding the SNP, and there were no insertions or deletions (indels) or additional variants within 30 or 20 bp of the targeted variable site, respectively. Additionally, we aligned 25 bp surrounding the target variable site to the genome using *bwa* (Burrows‐Wheeler Aligner; Li & Durbin, 2009) to determine whether the designable SNPs mapped uniquely to the red‐throated loon reference genome and filtered out those that mapped to multiple locations across the genome. A final subset of 192 SNPs was converted into SNPtype Assays (Fluidigm Inc.) specifically for population assignment in additional breeding individuals as well as samples collected from wintering sites and migratory stopover sites.

The Fluidigm Corporation EP1™ Genotyping System was used to screen 452 individuals at 192 SNPs in batches of 94 individuals per run with two non‐template controls. To ensure amplification of low quality or low concentration DNA from feathers, an initial preamplification step (von Thaden et al., 2017) was performed according to the manufacturer's protocol using a primer pool containing 96 un‐labeled locus‐specific SNP type primers. PCR products were diluted 1:10 and re‐amplified using fluorescently labeled allele‐specific primers. The results were imaged on an EP1 Array Reader, and alleles were called using Fluidigm's automated Genotyping Analysis Software (Fluidigm Inc.) with a confidence threshold of 90%. In addition, all SNP calls were visually inspected and any calls that did not fall clearly into one of three clusters—heterozygote or either homozygote cluster— were removed from the analysis. As DNA quality can affect call accuracy, a stringent quality filter was also employed; we excluded SNPs with greater than 20% missing calls. The resulting SNP assay set included 158 variants for common loons. Then, we used this SNP assay set to screen 200 additional breeding samples from across the breeding locations in the United States and Canada (Table S2). We also screened 252 nonbreeding birds on wintering areas and migration routes in order to assign them back to their respective conservation units.

### 2.7 Genetic screening and building the spatial map

To build the spatial map of genetic variation, we combined genotype data at the 158 loci described above from all breeding samples that were genotyped by either RAD‐PE or Fluidigm. Samples with missing genotypes at more than 10% of SNPs were removed from our analyses of population structure before constructing the spatial map. To assess population structure across the breeding range, we used the admixture model in *STRUCTURE* (version 2.3.4; (Pritchard et al., 2000), a model‐based clustering method. We implemented the *locprior* model that uses sampling locations as prior information, correlated allele frequencies, a burn‐in period of 50,000, and total run length of 150,000. We ran five iterations of each assumed number of genetic clusters (*K*), where *K* ranged from 1:7 (Pritchard et al., 2000). Results are shown in Figure S3.

Posterior probability of group membership estimates from *STRUCTURE* was visualized as transparency levels of different colors overlaid upon a base map from Natural Earth (naturalearthdata.com) and clipped to the common loon breeding range using a shapefile (NatureServe, 2012), making use of the R packages *SP*, *RGDAL*, and *RASTER* (Bivand et al., 2013, 2017; Hijmans, 2017). Thus, within each distinguishable group, the transparency of colors is scaled so that the highest posterior probability of membership in the group according to *STRUCTURE* is opaque and the smallest is transparent. This creates the spatially explicit map of genomic clustering, or genoscape of a species (Figure 1).

### 2.8 Baseline conservation units and accuracy of assignment

Accuracy of individual assignment analyses was evaluated for five of the six conservation units using a double cross‐validation approach (Anderson, 2010; Waples, 2010). The samples we used to assess assignment accuracy were new samples that we genotyped on the Fluidigm platform and not the original RAD‐PE genotyped samples that we used for delineation of the conservation units, estimation of allele frequencies and selection of the best loci to detect differences among the identified conservation units. We implemented the cross‐validation in *RUBIAS* (Moran & Anderson, 2018), a Bayesian hierarchical genetic identification approach that accounts for population structure and differences in the number of populations grouped into baseline conservation units. The self‐assessment function in *RUBIAS* tests the accuracy of assignment by assigning individuals in the reference back to the collections in the reference using a *leave*‐*one*‐*out* cross‐validation approach. Accuracy is defined as the proportion of individuals from known conservation units that are assigned back to the correct conservation unit. Individuals were defined as assigned to the correct conservation unit if they had a > 0.8 posterior probability of assignment to the unit encompassing the geographic location in which they were sampled. Because we lacked additional samples from eastern Canada with which to assess accuracy of assignment, we also ran a less rigorous cross‐validation in RUBIAS using both the additional Fluidigm genotyped samples and the original RAD‐PE genotyped samples.

### 2.9 Assignment of unknown migratory and wintering birds

Nonbreeding individuals of unknown origin (Table S3) were assigned to conservation units using *RUBIAS* (Moran & Anderson, 2018). The unknowns were either processed as a group with no location information or processed with location about the state from which they were collected. We saw no difference in assignment under these two strategies. We report the assignment of 142 of 164 wintering individuals (individuals with a posterior probability >0.8 of being assigned to a designated conservation unit) and the proportion of individuals assigned to conservation units of each migratory stopover site. We also examined relatedness among the migrating and wintering loons. Because these samples were only genotyped at 158 SNPs, we calculated the Lynch and Ritland (Lynch & Ritland, 1999) estimator in GenAlEx 6.503 (Peakall & Smouse, 2012).

### 2.10 Demographic trends within conservation units

We used Breeding Bird Survey (BBS) data (Sauer et al., 2017) to assess demographic trends within each conservation unit. Using *ARCGIS*, we constructed polygons of the conservation units and identified which BBS surveys fall within each conservation unit. We assigned digitized routes to the conservation unit that contained the majority of the route. We assigned nondigitized routes to the unit containing the starting point for that route. We only used data from standard surveys that pass basic quality standards (run type = 1) as set by BBS. We further filtered to include only those surveys for which both starting and ending wind conditions were <=2 on the Beaufort scale and for which both starting and ending sky conditions were <=2 (no fog, smoke or precipitation). Note that inclusion of surveys conducted in poorer but acceptable weather conditions did not alter abundance trends significantly.

## 3 RESULTS

We extracted 812 samples for use in ddRAD (156) and RAD‐PE (204) sequencing and Fluidigm assays (452). The samples were of several types: blood (594), blood dots (72), feathers (100), and other tissues (muscle or skin, 46). Samples from breeding birds (560) were from blood (497) or blood dots (63). Wintering samples (252) came from the remaining blood (97) and blood dot (9) samples and from the 146 feather and tissue samples listed above.

### 3.1 Genotyping success

In our initial ddRAD sequencing effort, 106 breeding individuals and 1852 SNPs passed our quality and minor allele frequency (MAF > 0.01) filters. RAD‐PE sequencing of 204 individuals from across the breeding range resulted in 180,198 SNPs passing our initial filters. After filtering for MAF > 0.03, relatedness, and additional missingness thresholds, our final dataset included 39,912 variants in 129 individuals. Most of the individuals removed from the RAD‐PE sample set were removed due to exceeding our threshold for missing genotypes. Twenty‐one individuals were removed due to relatedness, 11 of which had at least one first degree relative in the sample. Ten of the individuals removed due to relatedness were from the New England conservation unit, eight from the Midwest, two from central Canada, and one from the Pacific Northwest unit. Of the 200 additional breeding individuals genotyped using Fluidigm SNPtype assays, 173 passed filtering. Combining these 173 with the 127 RAD‐PE samples that passed filtering for the sites used in the Fluidigm assay gave us 300 individuals with which to define conservation units, build the spatial map, and conduct STRUCTURE analysis. Of 252 nonbreeding individuals genotyped using Fluidigm SNPtype assays, 164 passed filtering and could be used to perform assignments. All of the breeding loons were sampled while alive; however, the wintering loons were a mix of living birds and carcasses. We were able to genotype 88% of samples from living birds and 49% of the samples we received from common loon carcasses.

### 3.2 Initial analysis of population structure

Our analyses rely on SNPs derived from two different reduced representation (RAD) sequencing efforts. Our initial analysis based on double‐digest RAD‐seq (ddRAD; Peterson et al., 2012) revealed a clear east‐west axis of genetic differentiation, but provided insufficient power to fully discriminate among populations spanning from British Columbia through the Midwest and into Quebec (Figure S1). However, this dataset was useful for identifying Alaska as a well‐differentiated population, particularly given that Alaska was not well sampled by our second RAD sequencing effort (RAD‐PE; Ali et al., 2016). The RAD‐PE dataset was based on a much larger sample of loci, spanned a similar geographic range, and was demonstrably more powerful for delineating conservation units. PCA based on the RAD‐PE dataset showed distinct clustering of samples from different geographic areas and support for six differentiated regional populations, excluding Alaska for which we did not have enough samples, and including New York which did not separate out in subsequent analyses (Figure S2). We used preliminary information about population structure from the two RAD sequencing efforts to calculate allele frequency differences among the identified regional populations and to choose the most highly divergent and therefore informative SNPs for the design of Fluidigm SNP assays and the genotyping a larger set of samples.

### 3.3 Conservation units and accuracy of assignment

**FIGURE 2:**
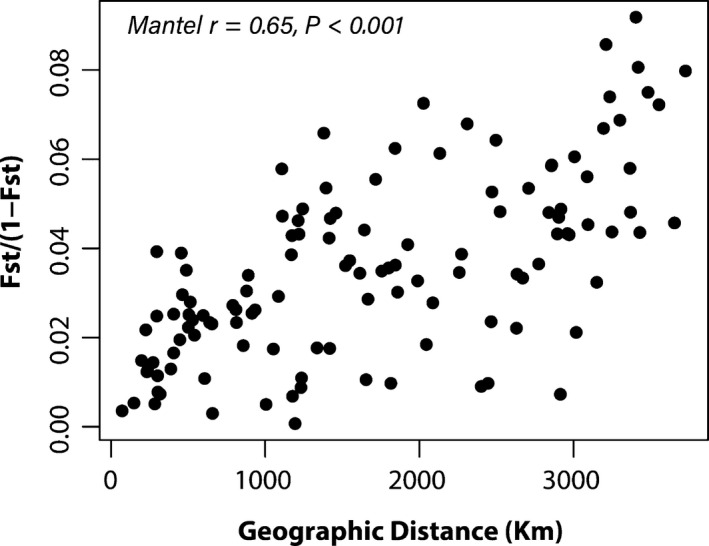
*F*_ST_ plotted by geographic distance for 16 sampling localities across the North American breeding range. *F*_ST_ values are calculated from the PE‐RAD‐seq data (39,912 SNPs in 129 individuals). *F*_ST_ values used in the figure are presented in Table S5

We identified six differentiated geographic clusters that we defined as conservation units. We define these as conservation units rather than populations because they were distinguished using the most divergent SNPs given the population structure elucidated by the two RAD‐seq efforts. From west to east, we designated these conservation units as Alaska, Pacific Northwest, central Canada, Midwest, eastern Canada, and New England (in which we include northern New York state) (Figure 1, Table 1). Genetic differentiation among conservation units indicates that the greatest genetic differentiation was found between the most eastern conservation units (New England and eastern Canada) and the most western conservation units (Pacific Northwest and Alaska), whereas central Canada and the Midwest were the least differentiated (Table 2). Interestingly, loons from the isolated Wyoming breeding grounds are genetically more similar to the central Canada conservation unit than the geographically closer Pacific Northwest conservation unit (Figure 1, Table S5). Analysis of *F*_ST_ among 16 smaller sampling regions shows a clear signal of isolation by distance (Figure 2, Table S5). Both cross‐validation and double cross‐validation of assignments indicated that over 90% of the samples from the Alaska, Pacific Northwest, and New England conservation units were assigned to the correct units, while samples from central Canada and the Midwest were correctly assigned at minimum rate of 82% and 89%, respectively (Table S4).

**TABLE 1:** Sampling Locations, codes, and identifying numbers used in Figure 1 and in Supplementary Tables and Figures

Figure 1 Map Number	Location code	Sampling region	Number of samples
1	AK	Alaska	24
2	BC	British Columbia	6
3	WA	Washington	7
4	MT	Montana	27
5	AB	Alberta	7
6	WY	Wyoming	15
7	SK	Saskatchewan	12
8	MT	Manitoba	5
9	ONT_W	Ontario, western	3
10	MN	Minnesota	2
11	WI1	Wisconsin 1	17
12	WI2	Wisconsin 2	5
13	MI1	Michigan 1	10
14	MI2	Michigan 2	20
15	MI3	Michigan 3	18
16	ONT_C	Ontario, central	2
17	ONT_E	Ontario, eastern	5
18	QB	Quebec	5
19	NY	New York	26
20	MA	Massachusetts	10
21	NH	New Hampshire	27
22	ME	Maine	40
23	NB	New Brunswick	6

**TABLE 2:** Pairwise *F*_ST_ values (upper triangle) and CI between five conservation units. Alaska, with only one sample in the RAD‐PE dataset, is excluded

Conservation unit	Pacific Northwest	Central Canada	Midwest	Eastern Canada	New England
Pacific Northwest		0.02	0.0448	0.0383	0.0624
Central Canada	0.0195–0.0206		0.0175	0.011	0.0364
Midwest	0.0437–0.0459	0.0169–0.0184		0.0093	0.0297
Eastern Canada	0.0372–0.0395	0.0102–0.0117	0.0082–0.0102		0.0184
New England	0.0611–0.0639	0.0357–0.037	0.029–0.0307	0.0176–0.0192	

We did not identify any first degree relatives (relatedness 0.25) among the sampled wintering loons. We did identify 77 pairs of individuals with relatedness scores between 0.125 and 0.196 (0.6% of all possible pairwise comparisons) and 1440 with relatedness scores between 0.0625 and 0.125 (10% of all possible pairwise comparisons).

### 3.4 Migratory connectivity

We found that migratory connectivity in common loons is somewhat diffuse (Figures 1b and S5). In all regions from which wintering or migratory birds were sampled, birds from at least two different breeding populations are present. Birds from the central Canada conservation unit and birds from the Midwest are widely represented in most or all of the wintering and migratory regions we sampled. Nonetheless, some interesting patterns emerge, some of which confirm data from satellite telemetry and banding recoveries (Evers et al., 2020). Birds breeding in New England tend to migrate only a short distance south and east to areas along the northeast coast of the United States, and individuals assigned to the Pacific Northwest conservation unit were only sampled along the southern California coast. The proportion of birds assigned to the central Canada conservation unit increased from east to west and the proportion of birds assigned to the Midwest population decreased from east to west. Birds with uncertain assignments (confidence <0.8, *n* = 23) were most often (*n* = 22) inferred to have mixed ancestry between central Canada and the Midwest. These included birds from Florida (six), Louisiana (five), Michigan (five), Ontario (three), California (one), Massachusetts (one), and Maine (one) (Table S3). An additional bird from California was inferred to have mixed ancestry between the Pacific Northwest and central Canada. We did not detect any migratory or wintering individuals from either the Alaskan conservation unit or the eastern Canada conservation unit. This lack of detection is more likely due to a lack of samples from wintering birds in appropriate offshore areas than it is due to a misassignment of existing wintering samples, as indicated by the high level of accuracy with which we were able to assign known individuals back to their populations of origin (Table S4).

Our data also suggest a previously undocumented migratory pathway from the Midwest (Figure S5). There is evidence that some breeding loons from the Midwest overwinter in coastal waters of California or Mexico.

### 3.5 Linking threats to conservation units

Migratory and wintering birds sampled from the Great Lakes during botulism outbreaks in 1999 and 2012 were identified as being from the Midwest and central Canada conservation units (Figure 1b). Birds sampled in Florida that were dead or moribund from unknown causes also came from either the Midwest or central Canada conservation units. Birds sampled along the northeast Coast of the United States that were dead or moribund from unknown causes were linked back to either the Midwest or New England conservation units. Birds found dead at solar array facilities in California were linked back to the Midwest and central Canada. Examination of BBS trends indicated that, despite such losses, breeding population demographic trends within each of the identified conservation units are generally stable (Figure S4).

## 4 DISCUSSION

A variety of techniques including banding, satellite tracking, and isotope analysis have been used in efforts to assess migratory connectivity (Bowlin et al., 2010; Clegg et al., 2003; Marra et al., 1998), but none can be used on carcasses. Our study illustrates the potential of using genomic approaches for assigning carcasses and other tissue samples of nonbreeding birds to their conservation units. This genomic approach draws on genetic mixed‐stock analysis developed for migratory fish species (Anderson et al., 2008, 2017; Waples et al., 2020) and has previously proven effective for assessing migratory connectivity in live birds (Bay et al., 2018; Ruegg et al., 2014); however, the ability to assign bird fatalities to conservation units is essential to understanding the impacts of disease and other risks on avian populations. Assigning carcasses to conservation units poses a significant challenge due to the degraded quality of DNA that can be obtained from carcasses, as DNA amplification and genotyping success is significantly influenced by the quality and quantity of the DNA that can be extracted (Taberlet et al., 1999). Our success rate when genotyping DNA from carcasses was comparable to results using shed feathers in other studies (Hogan et al., 2008). Therefore, we were able to link migrating and wintering birds to these conservation units using DNA sampled not only from live birds, but also using low quality DNA from carcasses. Being able to genotype birds that die in or *en route* to or from wintering grounds and identify their conservation unit of origin enhances our ability to predict the impacts of threats beyond the breeding grounds to population trends in those units.

We found that despite being a highly mobile migratory species, common loons show moderate population structure across their breeding grounds in North America, allowing the delineation of six genetically differentiated conservation units. We also found that genetic structure across North America is the result of an east to west pattern of isolation by distance. Consistent with the overall stability of the North American loon population (Evers et al., 2020; Sauer et al., 2017), our assessment is that breeding population demographic trends within each of these units are generally stable, although the stability in some regions is likely the result of intensive conservation efforts (Evers et al., 2020). While stable and increasing breeding populations have likely resulted from the more intensive conservation efforts in the southern part of the loon's breeding range (i.e., northern United States), there is now evidence that long‐term stability in Canada is being jeopardized by adverse impacts of mercury pollution and acid rain (e.g., an annual decline of 0.10 fledged chick/territorial has been observed over the past 40 years in Ontario; Bianchini et al., 2020). Our analysis provides a baseline for tracking further changes within these units, which is particularly salient considering recent evidence of declining populations in Ontario and across Canada (Bianchini et al., 2020; Tozer et al., 2013) and projected impacts from climate change and other diverse threats (Evers et al., 2020).

It is well known that diseases such as botulism may lead to mortality in water birds including loons (Evers, 2007; Kenow et al., 2018). We found that the majority of loons sampled in our study that were killed by botulism in the Great Lakes were from with the Midwest conservation unit, within which much of the Great Lakes lie. It is also notable that loons from the central Canada conservation unit made up a large portion of loons collected during botulism die‐offs, indicating this is an important staging area for multiple conservation units. Botulism outbreaks tend to impact migrating loons rather than breeding loons because the outbreaks occur during the autumn (October, November) when the Great Lakes are inundated with migrating loons from across Canada.

Climate change could exacerbate botulism risks to Midwest and central Canada loons as increasing lake temperatures are expected to increase the risk of botulism outbreaks (Lafrancois et al., 2011; Mooij et al., 2005) just as it increases bioavailable mercury levels and the risk of cyanobacteria outbreaks (Edmonds et al., 2012; Evers et al., 2020; Schindler, 2009). These same two populations are also impacted by solar arrays in the California desert, possibly due to what is called a “lake effect” in which birds are hypothesized to mistake the panels for water and attempt to land, resulting in either collision or stranding (Horváth et al., 2009; Walston et al., 2016), but further research is needed to assess the validity of this hypothesis.

One surprising finding is that a small, isolated region in northwestern Wyoming clustered with the central Canada conservation unit rather than with the Pacific Northwest unit to which it is geographically closer. This suggests that Wyoming loons are possibly a relic breeding population created from the retreat of glaciers across the western North American and the subsequent creation of nesting lakes (Cameron, 1922). Several studies have investigated the role of the Pleistocene glaciation in speciation and contemporary population structure of North American birds (Dohms et al., 2017; Johnson & Cicero, 2004; Zink, 1996) and several molecular studies have identified divergent lineages corresponding to lineages west and east of the Rocky Mountains in songbird species (Clegg et al., 2003; Lovette et al., 2004; Milot et al., 2000). One hypothesis is that the southern refugia of breeding common loons were associated with two glacial sheets (Cordilleran and Keewatin). While breeding loon populations in Montana, Washington and British Colombia may have been associated with the retreat of the Cordilleran ice sheet (based on its southernmost geographic extent), the Wyoming breeding loon population was perhaps associated with the Keewatin ice sheet, which retreated into central Canada. This finding is important to consider for conservation and restoration efforts of the Wyoming breeding loon population. Its genetic isolation has likely been over 10,000 years, and as it is the rarest breeding bird in Wyoming, the population's uniqueness becomes increasingly important for local conservation efforts.

We did not detect any wintering or migrating birds from the Alaskan or eastern Canadian conservation units. This is unlikely to be a failure of the assignment assay but is more likely to be due to a lack of samples originating from those areas. It is known that part of the Alaskan conservation unit migrates only a short distance to Alaskan coastal waters (BRI unpublished data based on band returns and satellite telemetry tracking), and we lacked samples of wintering birds from this area and coastal Pacific Northwest generally. The migratory behavior of Alaskan birds may mirror the short‐distance migratory behavior we observed in birds from the New England conservation unit. Our failure to detect loons migrating from the eastern Canada conservation unit is most likely because we lack samples from the region to which they are known to migrate. Banding returns indicate significant use of the North Carolina coast by loons from this conservation unit (Evers et al., 2020) and we have no samples from this wintering area. Finally, samples collected from California suggest the existence of a previously unrecognized migration pathway for breeding common loons from the Midwest to the California coast and the Gulf of California which could require loons to migrate across the Rockies. Further efforts are needed to better understand the magnitude of the population using this migratory route as it does not align with current tracking studies based on banding and satellite telemetry and would have significant ramifications for oil spill determinations of loon‐years‐lost and where to restore them (Evers et al., 2019).

In summary, our results show the utility of high‐resolution genetic data to inform management of migratory birds under a range of existing anthropogenic threats, and more saliently, future scenarios of increasing toxin threats (e.g., botulism type E and cyanobacteria) due to climate change. In addition, our detection of a possible transcontinental migration route was only possible because this approach allows efficient and cost‐effective screening of a large number of samples. Our current analysis was limited to 158 diagnostic genetic markers, but future research will reveal the extent to which additional genetic markers and more extensive sampling would allow us to further delineate structure in the loon population and increase the resolution of assignments of birds to conservation units.

## CONFLICT OF INTEREST

The authors declare no conflict of interest.

## Supporting information

Supplementary Material

## Data Availability

Genotypes and metadata for samples used in the study are available on Dryad: https://doi.org/10.5068/D1DD4F.
