# Supplementary material for: Leveraging genomics to understand threats to migratory birds
Source: Evol Appl. 2021 Apr 10;14(6):1646–58. doi: 10.1111/eva.13231 (PMC8210798; doi:10.1111/eva.13231)
Supplement: Supplementary file 1 — Supplementary Material [file EVA-14-1646-s001.docx]

**Supplementary Methods – ddRAD**

In a preliminary effort to characterize population structure, we employed a double digest RAD sequencing method detailed in (DaCosta & Sorenson, 2014) and assessed its utility in detecting fine scale population structure. For this paper, we restricted our analysis to 106 breeding birds (Table S1). For each sample, we double-digested 1.0 µg of genomic DNA with high fidelity versions of the *SbfI* and *EcoRI* restriction enzymes (New England Biolabs), and then ligated indexed sequencing adapters to each sample. Single end, 101 base pair reads were processed using the computational pipeline described by DaCosta and Sorenson (2014; https://github.com/BU-RAD-seq/ddRAD-seq-Pipeline). To obtain a set of high-quality SNPs for principal components analysis (PCA), we reduced the data set to 1,634 putative single-copy autosomal loci with one or more polymorphisms and with unambiguous genotypes for at least 90% of the 106 samples. After excluding rare alleles (< 1% frequency), the data set included 1,852 bi-allelic polymorphisms (SNPs or indels) at 1634 loci (RAD loci were ~ 97 bp in length).

**Supplementary Tables and Figures**

Table S1. Sampling locations for 106 North American breeding loons included in the ddRAD-seq data set.

| **SampleID** | **Country** | **State/Prov** | **sex** |
| --- | --- | --- | --- |
| 986 | USA | Maine | M |
| 1005 | USA | Maine | M |
| 1006 | USA | Maine | F |
| 1007 | USA | Maine | M |
| 1008 | USA | Maine | F |
| G018 | USA | Maine | M |
| G019 | USA | Maine | M |
| G022 | USA | Maine | F |
| G024 | USA | Maine | F |
| G101 | USA | Maine | M |
| 982 | USA | Massachusetts | M |
| 983 | USA | Massachusetts | M |
| 984 | USA | Massachusetts | M |
| G001 | Canada | New_Brunswick | F |
| G011 | Canada | New_Brunswick | M |
| G012 | Canada | New_Brunswick | F |
| G014 | Canada | New_Brunswick | M |
| G016 | Canada | New_Brunswick | M |
| 1030 | USA | New_Hampshire | F |
| 1031 | USA | New_Hampshire | M |
| G031 | USA | New_Hampshire | F |
| G032 | USA | New_Hampshire | F |
| G033 | USA | New_Hampshire | F |
| G034 | USA | New_Hampshire | M |
| G045 | USA | New_Hampshire | F |
| 1046 | USA | New_York | F |
| 1050 | USA | New_York | M |
| 1051 | USA | New_York | F |
| 1055 | USA | New_York | F |
| 1056 | USA | New_York | F |
| 1057 | USA | New_York | F |
| 1058 | USA | New_York | M |
| G037 | USA | New_York | M |
| G038 | USA | New_York | F |
| G041 | USA | New_York | F |
| G009 | USA | Nova_Scotia | M |
| G010 | Canada | Nova_Scotia | M |
| G025 | Canada | Quebec | M |
| G027 | Canada | Quebec | F |
| G028 | Canada | Quebec | M |
| G035 | Canada | Quebec | M |
| G036 | Canada | Quebec | M |
| G098 | Canada | Quebec | F |
| G107 | USA | Michigan | M |
| G108 | USA | Michigan | F |
| G073 | Canada | Ontario_C | M |
| G076 | Canada | Ontario_C | F |
| G088 | Canada | Ontario_C | M |
| G074 | Canada | Ontario_E | F |
| G075 | Canada | Ontario_E | M |
| G077 | Canada | Ontario_E | M |
| G078 | Canada | Ontario_E | M |
| G090 | Canada | Ontario_E | M |
| G095 | Canada | Ontario_E | F |
| G079 | Canada | Ontario_W | F |
| G080 | Canada | Ontario_W | M |
| G086 | Canada | Ontario_W | M |
| G094 | Canada | Ontario_W | M |
| G096 | Canada | Ontario_W | M |
| G097 | Canada | Ontario_W | F |
| G116 | USA | Wisconsin | F |
| G117 | USA | Wisconsin | F |
| G118 | USA | Wisconsin | M |
| G119 | USA | Wisconsin | F |
| G051 | Canada | Alberta | F |
| G052 | Canada | Alberta | M |
| G070 | Canada | Alberta | F |
| G071 | Canada | Alberta | M |
| G072 | Canada | Alberta | F |
| G082 | Canada | Alberta | M |
| G083 | Canada | Alberta | M |
| G084 | Canada | Alberta | F |
| G048 | Canada | Manitoba | M |
| G057 | Canada | Manitoba | F |
| G058 | Canada | Manitoba | M |
| G060 | Canada | Manitoba | M |
| G062 | Canada | Manitoba | M |
| G063 | Canada | Manitoba | M |
| G065 | Canada | Manitoba | F |
| G068 | Canada | Manitoba | F |
| G005 | USA | Montana | M |
| G006 | USA | Montana | M |
| G007 | USA | Montana | F |
| G053 | Canada | Saskatchewan | M |
| G054 | Canada | Saskatchewan | F |
| G056 | Canada | Saskatchewan | M |
| G064 | Canada | Saskatchewan | M |
| G066 | Canada | Saskatchewan | M |
| G067 | Canada | Saskatchewan | M |
| G069 | Canada | Saskatchewan | F |
| G081 | Canada | Saskatchewan | F |
| 967 | USA | Alaska | F |
| 968 | USA | Alaska | M |
| 970 | USA | Alaska | M |
| 971 | USA | Alaska | F |
| 972 | USA | Alaska | M |
| G040 | USA | Alaska | F |
| G042 | USA | Alaska | M |
| G099 | USA | Alaska | M |
| G046 | Canada | British Columbia | M |
| G047 | Canada | British Columbia | M |
| 1061 | USA | Washington | M |
| 1062 | USA | Washington | M |
| 1063 | USA | Washington | M |
| 1589 | USA | Washington | M |
| G043 | USA | Washington | M |
|  |  |  |  |
|  |  |  |  |
|  |  |  |  |
|  |  |  |  |
|  |  |  |  |
|  |  |  |  |

Table S2. Breeding bird samples used in RAD=PE, *STRUCTURE* analysis and for creation of the genoscape (spatial analysis). The two italicized samples from RAD-PE did not pass filters for use in the genoscape.

| Sample ID | Country | State/  Prov | Pop  Num | Lat | Long | Genotyped by Fluidigm | Genotyped by RAD-PE | Conservation Unit |
| --- | --- | --- | --- | --- | --- | --- | --- | --- |
| A02962 | USA | AK | 1 | 60.64 | -150.88 | 1 | 0 | Alaska |
| A02969 | USA | AK | 1 | 60.69 | -150.79 | 1 | 0 | Alaska |
| A02963 | USA | AK | 1 | 60.7 | -150.78 | 1 | 0 | Alaska |
| A02976 | USA | AK | 1 | 60.7 | -150.78 | 1 | 0 | Alaska |
| A02972 | USA | AK | 1 | 60.69 | -150.77 | 1 | 0 | Alaska |
| A03284 | USA | AK | 1 | 60.69 | -150.77 | 1 | 0 | Alaska |
| A03025 | USA | AK | 1 | 60.71 | -150.71 | 1 | 0 | Alaska |
| A03286 | USA | AK | 1 | 60.71 | -150.71 | 1 | 0 | Alaska |
| A02968 | USA | AK | 1 | 60.52 | -150.4 | 1 | 0 | Alaska |
| A02971 | USA | AK | 1 | 60.52 | -150.4 | 1 | 0 | Alaska |
| A03020 | USA | AK | 1 | 60.51 | -150.37 | 1 | 0 | Alaska |
| A03024 | USA | AK | 1 | 60.47 | -150.35 | 1 | 0 | Alaska |
| GI501p | USA | AK | 1 | 60.49 | -150.33 | 0 | 1 | Alaska |
| A02965 | USA | AK | 1 | 60.47 | -150.32 | 1 | 0 | Alaska |
| A03288 | USA | AK | 1 | 60.47 | -150.32 | 1 | 0 | Alaska |
| A03021 | USA | AK | 1 | 60.45 | -150.31 | 1 | 0 | Alaska |
| A03022 | USA | AK | 1 | 60.52 | -150.21 | 1 | 0 | Alaska |
| A02970 | USA | AK | 1 | 61.53 | -150 | 1 | 0 | Alaska |
| A02964 | USA | AK | 1 | 61.58 | -149.86 | 1 | 0 | Alaska |
| A02975 | USA | AK | 1 | 61.58 | -149.86 | 1 | 0 | Alaska |
| A02978 | USA | AK | 1 | 61.55 | -149.82 | 1 | 0 | Alaska |
| A02973 | USA | AK | 1 | 61.55 | -149.18 | 1 | 0 | Alaska |
| A03023 | USA | AK | 1 | 61.55 | -149.18 | 1 | 0 | Alaska |
| A03285 | USA | AK | 1 | 61.55 | -149.18 | 1 | 0 | Alaska |
| K00-02219 | CAN | BC | 2 | 49.87 | -123.56 | 0 | 1 | Pacific NW |
| 0938-78708 | CAN | BC | 2 | 50.56 | -122.47 | 0 | 1 | Pacific NW |
| 0938-03369 | CAN | BC | 2 | 50.54 | -122.45 | 0 | 1 | Pacific NW |
| A03006 | USA | WA | 3 | 45.6 | -122.4 | 1 | 0 | Pacific NW |
| A03004 | USA | WA | 3 | 42.27 | -121.76 | 1 | 0 | Pacific NW |
| 0938-78704 | CAN | BC | 2 | 51.53 | -120.77 | 0 | 1 | Pacific NW |
| 0938-78707 | CAN | BC | 2 | 51.3 | -120.54 | 0 | 1 | Pacific NW |
| 0938-78705 | CAN | BC | 2 | 51.25 | -120.39 | 0 | 1 | Pacific NW |
| A02999 | USA | WA | 3 | 48.8 | -119.04 | 1 | 0 | Pacific NW |
| NARL002057 | USA | WA | 3 | 48.51 | -118.84 | 0 | 1 | Pacific NW |
| A02998 | USA | WA | 3 | 48.52 | -118.81 | 1 | 0 | Pacific NW |
| NARL002056 | USA | WA | 3 | 48.52 | -118.81 | 0 | 1 | Pacific NW |
| NARL002058 | USA | WA | 3 | 48.9 | -118.14 | 0 | 1 | Pacific NW |
| 1118-16288 | USA | MT | 4 | 48.08 | -115.18 | 0 | 1 | Pacific NW |
| A02980 | USA | MT | 4 | 48.05 | -115.11 | 1 | 0 | Pacific NW |
| A02985 | USA | MT | 4 | 48.05 | -115.11 | 1 | 0 | Pacific NW |
| 0669-20501 | USA | MT | 4 | 48.03 | -115.07 | 1 | 0 | Pacific NW |
| 0938-44752 | USA | MT | 4 | 48.03 | -115.07 | 0 | 1 | Pacific NW |
| 0938-44758 | USA | MT | 4 | 48.56 | -115.07 | 0 | 1 | Pacific NW |
| 0938-44772 | USA | MT | 4 | 48.56 | -115.07 | 0 | 1 | Pacific NW |
| A02988 | USA | MT | 4 | 48.73 | -114.85 | 1 | 0 | Pacific NW |
| A02995 | USA | MT | 4 | 48.73 | -114.85 | 1 | 0 | Pacific NW |
| A02987 | USA | MT | 4 | 48.7 | -114.81 | 1 | 0 | Pacific NW |
| 669-217-40 | USA | MT | 4 | 48.7 | -114.8 | 0 | 1 | Pacific NW |
| 669-217-41 | USA | MT | 4 | 48.7 | -114.8 | 0 | 1 | Pacific NW |
| K00-02229 | CAN | AB | 5 | 52.75 | -114.74 | 1 | 0 | Central Canada |
| K00-02230 | CAN | AB | 5 | 52.75 | -114.74 | 0 | 1 | Central Canada |
| K08-21084 | CAN | AB | 5 | 52.75 | -114.74 | 0 | 1 | Central Canada |
| K08-21108 | CAN | AB | 5 | 52.75 | -114.74 | 0 | 1 | Central Canada |
| K09-24171 | CAN | AB | 5 | 52.75 | -114.74 | 0 | 1 | Central Canada |
| L99-81899 | CAN | AB | 5 | 52.75 | -114.74 | 0 | 1 | Central Canada |
| L99-81905 | CAN | AB | 5 | 52.75 | -114.74 | 0 | 1 | Central Canada |
| A02982 | USA | MT | 4 | 48.19 | -114.63 | 1 | 0 | Pacific NW |
| A02984 | USA | MT | 4 | 48.19 | -114.63 | 1 | 0 | Pacific NW |
| A02994 | USA | MT | 4 | 48.19 | -114.63 | 1 | 0 | Pacific NW |
| A02983 | USA | MT | 4 | 48.58 | -114.61 | 1 | 0 | Pacific NW |
| 669-217-47 | USA | MT | 4 | 48.14 | -114.59 | 0 | 1 | Pacific NW |
| 0938-44682 | USA | MT | 4 | 48.21 | -114.58 | 0 | 1 | Pacific NW |
| 0938-44780 | USA | MT | 4 | 48.21 | -114.58 | 0 | 1 | Pacific NW |
| 0669-20548 | USA | MT | 4 | 48.22 | -114.58 | 0 | 1 | Pacific NW |
| 0938-44796 | USA | MT | 4 | 48.9 | -114.42 | 0 | 1 | Pacific NW |
| 1058-00619 | USA | MT | 4 | 48.94 | -114.42 | 0 | 1 | Pacific NW |
| A02981 | USA | MT | 4 | 48.39 | -114.31 | 1 | 0 | Pacific NW |
| A02989 | USA | MT | 4 | 47.89 | -114.11 | 1 | 0 | Pacific NW |
| A02990 | USA | MT | 4 | 47.89 | -114.11 | 1 | 0 | Pacific NW |
| 1118-16285 | USA | MT | 4 | 47.7 | -113.77 | 0 | 1 | Pacific NW |
| A02986 | USA | MT | 4 | 47.33 | -113.59 | 1 | 0 | Pacific NW |
| 0968-87740 | USA | WY | 6 | 44.06 | -111.04 | 1 | 0 | Central Canada |
| 1058-00606 | USA | WY | 6 | 44.06 | -111.04 | 0 | 1 | Central Canada |
| 1058-00607 | USA | WY | 6 | 44.07 | -111.04 | 0 | 1 | Central Canada |
| 1058-00608 | USA | WY | 6 | 44.07 | -111.04 | 1 | 0 | Central Canada |
| 1058-00638 | USA | WY | 6 | 44.17 | -111.01 | 1 | 0 | Central Canada |
| 0938-78701 | USA | WY | 6 | 44.11 | -110.95 | 0 | 1 | Central Canada |
| 1118-15936 | USA | WY | 6 | 44.11 | -110.95 | 1 | 0 | Central Canada |
| 1058-00609 | USA | WY | 6 | 44.31 | -110.63 | 0 | 1 | Central Canada |
| 0938-78844 | USA | WY | 6 | 43.96 | -110.62 | 1 | 0 | Central Canada |
| 0938-78847 | USA | WY | 6 | 43.96 | -110.62 | 1 | 0 | Central Canada |
| 0938-78887 | USA | WY | 6 | 43.96 | -110.62 | 1 | 0 | Central Canada |
| 1118-15937 | USA | WY | 6 | 43.96 | -110.62 | 1 | 0 | Central Canada |
| *1058-00610* | *USA* | *WY* | *6* | *43.96* | *-110.62* | *0* | *1* | *Central Canada* |
| 1058-00611 | USA | WY | 6 | 44.75 | -110.59 | 0 | 1 | Central Canada |
| 1058-00648 | USA | WY | 6 | 44.36 | -110.55 | 1 | 0 | Central Canada |
| 0968-87747 | USA | WY | 6 | 44.38 | -110.39 | 0 | 1 | Central Canada |
| 1118-15998 | CAN | SK | 7 | 54.46 | -109.05 | 0 | 1 | Central Canada |
| 0938-03492 | CAN | SK | 7 | 54.4 | -108.96 | 0 | 1 | Central Canada |
| 0938-78822 | CAN | SK | 7 | 54.41 | -108.96 | 0 | 1 | Central Canada |
| 1118-15954 | CAN | SK | 7 | 54.42 | -108.92 | 0 | 1 | Central Canada |
| 1118-15955 | CAN | SK | 7 | 54.43 | -108.81 | 0 | 1 | Central Canada |
| 1118-15953 | CAN | SK | 7 | 54.42 | -108.77 | 0 | 1 | Central Canada |
| K08-20979 | CAN | SK | 7 | 54.17 | -104.78 | 0 | 1 | Central Canada |
| K08-21013 | CAN | SK | 7 | 54.17 | -104.7 | 0 | 1 | Central Canada |
| K08-20974 | CAN | SK | 7 | 53.99 | -104.68 | 0 | 1 | Central Canada |
| K08-20975 | CAN | SK | 7 | 54.27 | -104.6 | 0 | 1 | Central Canada |
| K08-21018 | CAN | SK | 7 | 54.27 | -104.56 | 0 | 1 | Central Canada |
| K08-21032 | CAN | SK | 7 | 54.79 | -102.85 | 0 | 1 | Central Canada |
| K08-21004 | CAN | MAN | 8 | 54.84 | -101.71 | 1 | 0 | Central Canada |
| K08-21030 | CAN | MAN | 8 | 54.84 | -101.71 | 0 | 1 | Central Canada |
| K08-20994 | CAN | MAN | 8 | 54.75 | -101.66 | 0 | 1 | Central Canada |
| K08-20981 | CAN | MAN | 8 | 54.76 | -101.57 | 0 | 1 | Central Canada |
| K08-21007 | CAN | MAN | 8 | 54.67 | -101.55 | 0 | 1 | Central Canada |
| 0938-06495 | USA | MN | 10 | 47.48 | -93.59 | 1 | 0 | Midwest |
| 0938-78716 | USA | MN | 10 | 47.48 | -93.59 | 1 | 0 | Midwest |
| A03430 | USA | WI1 | 11 | 45.69 | -91.71 | 1 | 0 | Midwest |
| A03118 | USA | WI1 | 11 | 45.69 | -91.7 | 1 | 0 | Midwest |
| A03112 | USA | WI2 | 12 | 46.38 | -91.53 | 1 | 0 | Midwest |
| A03113 | USA | WI1 | 11 | 45.22 | -91.3 | 1 | 0 | Midwest |
| NXX-46113 | CAN | ONT_W | 9 | 53.75 | -89.92 | 0 | 1 | Central Canada |
| A03119 | USA | WI1 | 11 | 45.63 | -89.78 | 1 | 0 | Midwest |
| A03434 | USA | WI1 | 11 | 45.63 | -89.78 | 1 | 0 | Midwest |
| A03122 | USA | WI1 | 11 | 45.75 | -89.7 | 1 | 0 | Midwest |
| A03433 | USA | WI1 | 11 | 45.75 | -89.7 | 1 | 0 | Midwest |
| A03427 | USA | WI1 | 11 | 45.62 | -89.66 | 1 | 0 | Midwest |
| A03289 | USA | WI1 | 11 | 45.74 | -89.66 | 1 | 0 | Midwest |
| A03429 | USA | WI1 | 11 | 45.77 | -89.61 | 1 | 0 | Midwest |
| A03432 | USA | WI1 | 11 | 45.77 | -89.61 | 1 | 0 | Midwest |
| A03117 | USA | WI1 | 11 | 45.78 | -89.58 | 1 | 0 | Midwest |
| A03428 | USA | WI1 | 11 | 45.81 | -89.58 | 1 | 0 | Midwest |
| A03114 | USA | WI1 | 11 | 45.84 | -89.58 | 1 | 0 | Midwest |
| Wi04-43820 | USA | WI2 | 12 | 46.03 | -89.56 | 0 | 1 | Midwest |
| Wi04-43821 | USA | WI2 | 12 | 46.03 | -89.56 | 1 | 0 | Midwest |
| Wi04-43823 | USA | WI2 | 12 | 46.03 | -89.56 | 0 | 1 | Midwest |
| Wi04-43824 | USA | WI2 | 12 | 46.03 | -89.56 | 0 | 1 | Midwest |
| A03431 | USA | WI1 | 11 | 45.78 | -89.49 | 1 | 0 | Midwest |
| L95-71354 | CAN | ONT_W | 9 | 48.84 | -89.37 | 0 | 1 | Central Canada |
| A03115 | USA | WI1 | 11 | 45.81 | -89.3 | 1 | 0 | Midwest |
| A03116 | USA | WI1 | 11 | 45.79 | -89.04 | 1 | 0 | Midwest |
| A03126 | USA | MI2 | 14 | 47.99 | -88.87 | 1 | 0 | Midwest |
| A03191 | USA | MI2 | 14 | 48.03 | -88.78 | 1 | 0 | Midwest |
| A03192 | USA | MI2 | 14 | 48.03 | -88.78 | 1 | 0 | Midwest |
| A03193 | USA | MI2 | 14 | 48.03 | -88.78 | 1 | 0 | Midwest |
| A03194 | USA | MI2 | 14 | 48.03 | -88.78 | 1 | 0 | Midwest |
| A03195 | USA | MI2 | 14 | 48.03 | -88.78 | 1 | 0 | Midwest |
| A03196 | USA | MI2 | 14 | 48.03 | -88.78 | 1 | 0 | Midwest |
| A03197 | USA | MI2 | 14 | 48.03 | -88.78 | 1 | 0 | Midwest |
| A03198 | USA | MI2 | 14 | 48.03 | -88.78 | 1 | 0 | Midwest |
| A03199 | USA | MI2 | 14 | 48.03 | -88.78 | 1 | 0 | Midwest |
| A03200 | USA | MI2 | 14 | 48.03 | -88.78 | 1 | 0 | Midwest |
| A03441 | USA | MI2 | 14 | 48.03 | -88.78 | 1 | 0 | Midwest |
| A03442 | USA | MI2 | 14 | 48.03 | -88.78 | 1 | 0 | Midwest |
| A03446 | USA | MI2 | 14 | 48.03 | -88.78 | 1 | 0 | Midwest |
| A03447 | USA | MI2 | 14 | 48.03 | -88.78 | 1 | 0 | Midwest |
| A03450 | USA | MI2 | 14 | 48.03 | -88.78 | 1 | 0 | Midwest |
| A03451 | USA | MI2 | 14 | 48.03 | -88.78 | 1 | 0 | Midwest |
| A03452 | USA | MI2 | 14 | 48.03 | -88.78 | 1 | 0 | Midwest |
| A03125 | USA | MI2 | 14 | 48.14 | -88.63 | 1 | 0 | Midwest |
| A03124 | USA | MI2 | 14 | 48.14 | -88.49 | 1 | 0 | Midwest |
| L95-71353 | CAN | ONT_W | 9 | 49.31 | -87.79 | 0 | 1 | Central Canada |
| *GiJv234Q1* | *USA* | *MI3* | *15* | *46.29* | *-85.95* | *0* | *1* | *Midwest* |
| GiJv240Q1 | USA | MI3 | 15 | 46.29 | -85.95 | 1 | 0 | Midwest |
| GiJv241Q1 | USA | MI3 | 15 | 46.29 | -85.95 | 1 | 0 | Midwest |
| GiJv243Q1 | USA | MI3 | 15 | 46.29 | -85.95 | 0 | 1 | Midwest |
| GiJv245Q1 | USA | MI3 | 15 | 46.29 | -85.95 | 0 | 1 | Midwest |
| GiJv246Q1 | USA | MI3 | 15 | 46.29 | -85.95 | 0 | 1 | Midwest |
| GiJv249Q1 | USA | MI3 | 15 | 46.29 | -85.95 | 0 | 1 | Midwest |
| GiJv250Q1 | USA | MI3 | 15 | 46.29 | -85.95 | 0 | 1 | Midwest |
| A03141 | USA | MI3 | 15 | 46.28 | -85.94 | 1 | 0 | Midwest |
| A03143 | USA | MI3 | 15 | 46.28 | -85.94 | 1 | 0 | Midwest |
| A03144 | USA | MI3 | 15 | 46.28 | -85.94 | 1 | 0 | Midwest |
| A03203 | USA | MI3 | 15 | 46.28 | -85.94 | 1 | 0 | Midwest |
| A03205 | USA | MI3 | 15 | 46.28 | -85.94 | 1 | 0 | Midwest |
| A03206 | USA | MI3 | 15 | 46.28 | -85.94 | 1 | 0 | Midwest |
| A03207 | USA | MI3 | 15 | 46.28 | -85.94 | 1 | 0 | Midwest |
| A03208 | USA | MI3 | 15 | 46.28 | -85.94 | 1 | 0 | Midwest |
| A03469 | USA | MI3 | 15 | 46.28 | -85.94 | 1 | 0 | Midwest |
| A03470 | USA | MI3 | 15 | 46.28 | -85.94 | 1 | 0 | Midwest |
| A03475 | USA | MI3 | 15 | 46.28 | -85.94 | 1 | 0 | Midwest |
| A03448 | USA | MI1 | 13 | 44.97 | -85.2 | 1 | 0 | Midwest |
| A03454 | USA | MI1 | 13 | 44.97 | -85.2 | 1 | 0 | Midwest |
| A03458 | USA | MI1 | 13 | 44.97 | -85.2 | 1 | 0 | Midwest |
| A03453 | USA | MI1 | 13 | 45 | -85.2 | 1 | 0 | Midwest |
| A03455 | USA | MI1 | 13 | 45 | -85.2 | 1 | 0 | Midwest |
| A03456 | USA | MI1 | 13 | 45 | -85.2 | 1 | 0 | Midwest |
| A03459 | USA | MI1 | 13 | 45 | -85.2 | 1 | 0 | Midwest |
| A03460 | USA | MI1 | 13 | 45 | -85.2 | 1 | 0 | Midwest |
| A03449 | USA | MI1 | 13 | 45.07 | -85.18 | 1 | 0 | Midwest |
| A03457 | USA | MI1 | 13 | 45.07 | -85.18 | 1 | 0 | Midwest |
| L94-69178 | CAN | ONT_C | 16 | 45.73 | -78.41 | 0 | 1 | Eastern Canada |
| NXX-24049 | CAN | ONT_C | 16 | 45.76 | -78.34 | 0 | 1 | Eastern Canada |
| NXX-39883 | CAN | ONT_E | 17 | 44.49 | -77.87 | 0 | 1 | Eastern Canada |
| L94-69175 | CAN | ONT_E | 17 | 44.27 | -77.52 | 0 | 1 | Eastern Canada |
| L94-69176 | CAN | ONT_E | 17 | 44.27 | -77.52 | 0 | 1 | Eastern Canada |
| L94-69180 | CAN | ONT_E | 17 | 44.27 | -77.52 | 0 | 1 | Eastern Canada |
| L94-68604 | CAN | ONT_E | 17 | 44.36 | -76.46 | 0 | 1 | Eastern Canada |
| 938-152-73 | CAN | QB | 18 | 45.85 | -76.45 | 0 | 1 | Eastern Canada |
| 938-033-19 | CAN | QB | 18 | 46.21 | -75.8 | 0 | 1 | Eastern Canada |
| 938-033-26 | CAN | QB | 18 | 46.21 | -75.8 | 0 | 1 | Eastern Canada |
| bear | CAN | QB | 18 | 46.21 | -75.8 | 0 | 1 | Eastern Canada |
| A03086 | USA | NY | 19 | 43.85 | -75.17 | 1 | 0 | New England |
| A03108 | USA | NY | 19 | 43.85 | -75.17 | 1 | 0 | New England |
| A03109 | USA | NY | 19 | 43.59 | -74.98 | 1 | 0 | New England |
| 0898-09854 | USA | NY | 19 | 44.21 | -74.94 | 0 | 1 | New England |
| 0938-61788 | USA | NY | 19 | 44.21 | -74.94 | 0 | 1 | New England |
| A03090 | USA | NY | 19 | 43.69 | -74.92 | 1 | 0 | New England |
| A03091 | USA | NY | 19 | 43.69 | -74.92 | 1 | 0 | New England |
| A03092 | USA | NY | 19 | 43.76 | -74.9 | 1 | 0 | New England |
| A03093 | USA | NY | 19 | 43.76 | -74.9 | 1 | 0 | New England |
| A03087 | USA | NY | 19 | 43.83 | -74.84 | 1 | 0 | New England |
| A03088 | USA | NY | 19 | 43.83 | -74.84 | 1 | 0 | New England |
| A03089 | USA | NY | 19 | 43.83 | -74.84 | 1 | 0 | New England |
| 0938-61770 | USA | NY | 19 | 44.15 | -74.82 | 0 | 1 | New England |
| 938-153-13 | USA | NY | 19 | 44.26 | -74.64 | 0 | 1 | New England |
| UnNY-14001 | USA | NY | 19 | 44.23 | -74.56 | 0 | 1 | New England |
| 938-153-12 | USA | NY | 19 | 43.44 | -74.48 | 0 | 1 | New England |
| A03102 | USA | NY | 19 | 44.02 | -74.27 | 1 | 0 | New England |
| A03104 | USA | NY | 19 | 43.99 | -74.24 | 1 | 0 | New England |
| 938-153-67 | USA | NY | 19 | 43.96 | -74.15 | 0 | 1 | New England |
| A03094 | USA | NY | 19 | 42.19 | -74.04 | 1 | 0 | New England |
| A03095 | USA | NY | 19 | 42.19 | -74.04 | 1 | 0 | New England |
| A03096 | USA | NY | 19 | 42.19 | -74.04 | 1 | 0 | New England |
| A03097 | USA | NY | 19 | 42.19 | -74.04 | 1 | 0 | New England |
| 0649-08826 | USA | NY | 19 | 43.54 | -74.01 | 0 | 1 | New England |
| A03099 | USA | NY | 19 | 44.48 | -73.85 | 1 | 0 | New England |
| A03100 | USA | NY | 19 | 44.48 | -73.85 | 1 | 0 | New England |
| 918-133-21 | CAN | QB | 18 | 47.18 | -73.26 | 0 | 1 | New England |
| 0938-44359 | USA | MA | 20 | 42.45 | -72.39 | 1 | 0 | New England |
| A03035 | USA | MA | 20 | 42.38 | -72.31 | 1 | 0 | New England |
| 0669-21928 | USA | MA | 20 | 42.49 | -72.31 | 0 | 1 | New England |
| 0938-44353 | USA | MA | 20 | 42.39 | -72.24 | 0 | 1 | New England |
| 1118-15911 | USA | MA | 20 | 42.39 | -72.24 | 0 | 1 | New England |
| 0938-66647 | USA | MA | 20 | 42.49 | -71.82 | 0 | 1 | New England |
| 1118-15259 | USA | NH | 21 | 43.45 | -71.81 | 0 | 1 | New England |
| 1118-16189 | USA | NH | 21 | 43.45 | -71.81 | 0 | 1 | New England |
| 0669-21930 | USA | MA | 20 | 42.39 | -71.74 | 0 | 1 | New England |
| A03036 | USA | MA | 20 | 42.39 | -71.72 | 1 | 0 | New England |
| A03070 | USA | NH | 21 | 43.72 | -71.6 | 1 | 0 | New England |
| 938-152-34 | USA | NH | 21 | 43.61 | -71.59 | 0 | 1 | New England |
| 0938-44710 | USA | NH | 21 | 43.74 | -71.55 | 0 | 1 | New England |
| 1118-15256 | USA | NH | 21 | 43.74 | -71.55 | 0 | 1 | New England |
| 938-063-50 | USA | NH | 21 | 43.74 | -71.53 | 0 | 1 | New England |
| A03062 | USA | NH | 21 | 43.76 | -71.53 | 1 | 0 | New England |
| A03063 | USA | NH | 21 | 43.76 | -71.53 | 1 | 0 | New England |
| A03064 | USA | NH | 21 | 43.76 | -71.53 | 1 | 0 | New England |
| A03065 | USA | NH | 21 | 43.76 | -71.53 | 1 | 0 | New England |
| A03077 | USA | NH | 21 | 43.76 | -71.53 | 1 | 0 | New England |
| 0898-09936 | USA | NH | 21 | 43.71 | -71.51 | 1 | 0 | New England |
| 0898-09097 | USA | NH | 21 | 43.73 | -71.5 | 1 | 0 | New England |
| 0559-61768 | USA | NH | 21 | 42.97 | -71.38 | 1 | 0 | New England |
| 1118-16200 | USA | NH | 21 | 42.97 | -71.38 | 0 | 1 | New England |
| A03074 | USA | NH | 21 | 43 | -71.37 | 1 | 0 | New England |
| 1118-15253 | USA | NH | 21 | 43.72 | -71.37 | 0 | 1 | New England |
| A03072 | USA | NH | 21 | 43.6 | -71.33 | 1 | 0 | New England |
| A03075 | USA | NH | 21 | 43.6 | -71.33 | 1 | 0 | New England |
| A03082 | USA | NH | 21 | 43.6 | -71.33 | 1 | 0 | New England |
| 1118-16196 | USA | NH | 21 | 43.68 | -71.29 | 0 | 1 | New England |
| A03066 | USA | NH | 21 | 43.21 | -71.25 | 1 | 0 | New England |
| 0669-55046 | USA | MA | 20 | 42.8 | -71.15 | 1 | 0 | New England |
| 0938-78852 | USA | MA | 20 | 42.8 | -71.15 | 0 | 1 | New England |
| 0938-44733 | USA | NH | 21 | 44.83 | -71.13 | 0 | 1 | New England |
| 938-064-62 | USA | NH | 21 | 44.77 | -71.04 | 0 | 1 | New England |
| 938-064-72 | USA | NH | 21 | 44.77 | -71.04 | 1 | 0 | New England |
| 938-308-99 | USA | NH | 21 | 44.77 | -71.04 | 0 | 1 | New England |
| 0938-78856 | USA | ME | 22 | 44.99 | -71.01 | 0 | 1 | New England |
| A03039 | USA | ME | 22 | 45 | -71.01 | 1 | 0 | New England |
| A03042 | USA | ME | 22 | 45 | -71.01 | 1 | 0 | New England |
| 0669-55026 | USA | ME | 22 | 45.09 | -70.99 | 0 | 1 | New England |
| A03043 | USA | ME | 22 | 45.14 | -70.98 | 1 | 0 | New England |
| A03053 | USA | ME | 22 | 45.14 | -70.98 | 1 | 0 | New England |
| A03040 | USA | ME | 22 | 44.77 | -70.9 | 1 | 0 | New England |
| A03047 | USA | ME | 22 | 44.77 | -70.9 | 1 | 0 | New England |
| 0649-08802 | USA | ME | 22 | 44.81 | -70.89 | 1 | 0 | New England |
| 1118-15976 | USA | ME | 22 | 44.93 | -70.88 | 0 | 1 | New England |
| 0938-06395 | USA | ME | 22 | 44.98 | -70.82 | 0 | 1 | New England |
| A03052 | USA | ME | 22 | 44.94 | -70.8 | 1 | 0 | New England |
| 0669-55025 | USA | ME | 22 | 43.74 | -70.74 | 1 | 0 | New England |
| 0669-55030 | USA | ME | 22 | 43.74 | -70.74 | 1 | 0 | New England |
| 0649-08897 | USA | ME | 22 | 45.35 | -70.7 | 0 | 1 | New England |
| 898-098-99 | USA | ME | 22 | 45.33 | -70.65 | 0 | 1 | New England |
| A03038 | USA | ME | 22 | 45.02 | -70.64 | 1 | 0 | New England |
| 898-099-31 | USA | ME | 22 | 44.93 | -70.63 | 0 | 1 | New England |
| A03060 | USA | ME | 22 | 45.32 | -70.63 | 1 | 0 | New England |
| 0669-55045 | USA | ME | 22 | 43.74 | -70.62 | 0 | 1 | New England |
| A03041 | USA | ME | 22 | 44.97 | -70.55 | 1 | 0 | New England |
| 0669-21938 | USA | ME | 22 | 43.97 | -70.5 | 0 | 1 | New England |
| 0669-55040 | USA | ME | 22 | 43.85 | -70.43 | 1 | 0 | New England |
| 0938-15148 | USA | ME | 22 | 43.98 | -70.43 | 0 | 1 | New England |
| 0938-78855 | USA | ME | 22 | 43.98 | -70.43 | 0 | 1 | New England |
| 1118-15967 | USA | ME | 22 | 43.87 | -70.41 | 0 | 1 | New England |
| 0938-26125 | USA | ME | 22 | 43.89 | -70.41 | 0 | 1 | New England |
| 0938-44727 | USA | ME | 22 | 43.89 | -70.4 | 1 | 0 | New England |
| 0669-55050 | USA | ME | 22 | 43.95 | -70.38 | 0 | 1 | New England |
| 0938-06438 | USA | ME | 22 | 43.95 | -70.38 | 0 | 1 | New England |
| 0938-66659 | USA | ME | 22 | 44.02 | -70.38 | 0 | 1 | New England |
| A03045 | USA | ME | 22 | 45.21 | -70.37 | 1 | 0 | New England |
| A03055 | USA | ME | 22 | 45.21 | -70.37 | 1 | 0 | New England |
| 1118-15996 | USA | ME | 22 | 43.93 | -70.36 | 1 | 0 | New England |
| 898-098-63 | USA | ME | 22 | 45.15 | -70.36 | 0 | 1 | New England |
| 1118-15973 | USA | ME | 22 | 45.21 | -70.3 | 0 | 1 | New England |
| 898-098-59 | USA | ME | 22 | 46.02 | -70.05 | 0 | 1 | New England |
| 898-098-55 | USA | ME | 22 | 44.42 | -68.63 | 0 | 1 | New England |
| A03287 | USA | ME | 22 | 44.5 | -68.60 | 1 | 0 | New England |
| 898-099-83 | USA | ME | 22 | 44.82 | -68.48 | 0 | 1 | New England |
| 1118-15956 | USA | ME | 22 | 44.35 | -68.38 | 0 | 1 | New England |
| 898-053-74 | CAN | NB | 23 | 45.56 | -67.01 | 0 | 1 | New England |
| 898-053-76 | CAN | NB | 23 | 45.56 | -67.01 | 1 | 0 | New England |
| 898-053-81 | CAN | NB | 23 | 45.01 | -66.96 | 0 | 1 | New England |
| 898-053-77 | CAN | NB | 23 | 45.16 | -66.79 | 0 | 1 | New England |
| 898-053-80 | CAN | NB | 23 | 45.42 | -66.71 | 0 | 1 | New England |
| 898-053-87 | CAN | NB | 23 | 45.25 | -66.26 | 0 | 1 | New England |

Table S3. Assignment to conservation units of samples from non-breeding birds. The 142 samples assigned with confidence (> 0.8) are in bold.

|  |  | **Assignment Confidence** | | | | | |  |  |  |
| --- | --- | --- | --- | --- | --- | --- | --- | --- | --- | --- |
| Sample ID | State/Prov | Alaska | Pacific Northwest | central Canada | Midwest | eastern Canada | New England | Lat | Long | Figure 1 Location |
| A03011 | CA | 0.00 | 0.00 | **1.00** | 0.00 | 0.00 | 0.00 | 35.36 | -120.9 | A |
| A03018 | CA | 0.00 | 0.00 | **1.00** | 0.00 | 0.00 | 0.00 | 35.36 | -120.9 | A |
| A03008 | CA | 0.00 | 0.00 | **1.00** | 0.00 | 0.00 | 0.00 | 35.36 | -120.9 | A |
| A03017 | CA | 0.00 | 0.00 | **1.00** | 0.00 | 0.00 | 0.00 | 35.36 | -120.9 | A |
| A02957 | CA | 0.00 | 0.00 | **0.90** | 0.10 | 0.00 | 0.00 | 35.36 | -120.9 | A |
| A03010 | CA | 0.00 | 0.00 | **1.00** | 0.00 | 0.00 | 0.00 | 35.36 | -120.9 | A |
| A03027 | CA | 0.00 | 0.00 | **1.00** | 0.00 | 0.00 | 0.00 | 35.36 | -120.9 | A |
| A03032 | CA | 0.00 | 0.00 | **0.99** | 0.01 | 0.00 | 0.00 | 35.36 | -120.9 | A |
| A02955 | CA | 0.00 | 0.00 | **1.00** | 0.00 | 0.00 | 0.00 | 35.36 | -120.9 | A |
| A03033 | CA | 0.00 | 0.00 | **1.00** | 0.00 | 0.00 | 0.00 | 35.36 | -120.9 | A |
| A03013 | CA | 0.00 | 0.00 | **0.98** | 0.02 | 0.00 | 0.00 | 35.36 | -120.9 | A |
| A03016 | CA | 0.00 | 0.00 | **1.00** | 0.00 | 0.00 | 0.00 | 35.36 | -120.9 | A |
| A03028 | CA | 0.00 | 0.00 | **0.99** | 0.01 | 0.00 | 0.00 | 35.36 | -120.9 | A |
| A03009 | CA | 0.00 | 0.00 | **1.00** | 0.00 | 0.00 | 0.00 | 35.36 | -120.9 | A |
| A03014 | CA | 0.00 | 0.00 | **1.00** | 0.00 | 0.00 | 0.00 | 35.36 | -120.9 | A |
| A03026 | CA | 0.00 | 0.00 | **1.00** | 0.00 | 0.00 | 0.00 | 35.36 | -120.9 | A |
| A03031 | CA | 0.00 | 0.00 | **0.97** | 0.03 | 0.00 | 0.00 | 35.36 | -120.9 | A |
| A02951 | CA | 0.00 | 0.00 | **0.99** | 0.01 | 0.00 | 0.00 | 35.36 | -120.9 | A |
| A03030 | CA | 0.00 | 0.00 | **1.00** | 0.00 | 0.00 | 0.00 | 35.36 | -120.9 | A |
| A02952 | CA | 0.00 | 0.00 | 0.07 | **0.93** | 0.00 | 0.00 | 35.36 | -120.9 | A |
| A03034 | CA | 0.00 | 0.00 | 0.12 | **0.88** | 0.00 | 0.00 | 35.36 | -120.9 | A |
| A03012 | CA | 0.00 | **1.00** | 0.00 | 0.00 | 0.00 | 0.00 | 35.36 | -120.9 | A |
| A02958 | CA | 0.00 | **1.00** | 0.00 | 0.00 | 0.00 | 0.00 | 35.36 | -120.9 | A |
| A03029 | CA | 0.00 | **1.00** | 0.00 | 0.00 | 0.00 | 0.00 | 35.36 | -120.9 | A |
| A02950 | CA | 0.00 | 0.32 | 0.59 | 0.09 | 0.00 | 0.00 | 35.36 | -120.9 |  |
| 16N1789 | CA | 0.00 | 0.00 | **0.95** | 0.05 | 0.00 | 0.00 | 36.07 | -119.0 | A |
| 15N2859 | CA | 0.00 | 0.00 | **0.89** | 0.11 | 0.00 | 0.00 | 35.72 | -115.4 | B |
| 16N0925 | CA | 0.00 | 0.00 | **1.00** | 0.00 | 0.00 | 0.00 | 35.72 | -115.4 | B |
| 16N4380 | CA | 0.00 | 0.00 | **0.82** | 0.18 | 0.00 | 0.00 | 35.72 | -115.4 | B |
| 15N2869 | CA | 0.00 | 0.00 | **0.99** | 0.01 | 0.00 | 0.00 | 35.72 | -115.4 | B |
| 14N1749 | CA | 0.00 | 0.00 | **0.98** | 0.02 | 0.00 | 0.00 | 35.72 | -115.4 | B |
| 17N03915 | CA | 0.00 | 0.00 | **1.00** | 0.00 | 0.00 | 0.00 | 35.72 | -115.4 | B |
| 16N0928 | CA | 0.00 | 0.00 | **1.00** | 0.00 | 0.00 | 0.00 | 35.72 | -115.4 | B |
| 17N03914 | CA | 0.00 | 0.00 | 0.03 | **0.97** | 0.00 | 0.00 | 35.72 | -115.4 | B |
| 18N01807 | CA | 0.00 | 0.00 | **1.00** | 0.00 | 0.00 | 0.00 | 33.71 | -114.8 | B |
| 17N02985 | CA | 0.00 | 0.00 | **0.97** | 0.03 | 0.00 | 0.00 | 33.65 | -114.7 | B |
| 18N01772 | CA | 0.00 | 0.00 | 0.45 | 0.55 | 0.00 | 0.00 | 33.65 | -114.7 |  |
| 16N2917 | CA | 0.00 | 0.00 | **0.88** | 0.12 | 0.00 | 0.00 | 33.13 | -114.5 | B |
| 16N3315 | CA | 0.00 | 0.00 | **1.00** | 0.00 | 0.00 | 0.00 | 33.13 | -114.5 | B |
| 18N01797 | CA | 0.00 | 0.00 | 0.14 | **0.86** | 0.00 | 0.00 | 33.13 | -114.5 | B |
| 14N1740 | CA | 0.00 | 0.00 | 0.11 | **0.89** | 0.00 | 0.00 | 33.13 | -114.5 | B |
| A03374 | LA | 0.00 | 0.00 | **0.89** | 0.11 | 0.00 | 0.00 | 30.29 | -91.23 | F |
| A03368 | LA | 0.00 | 0.00 | 0.01 | **0.99** | 0.00 | 0.00 | 30.29 | -91.23 | F |
| A03365 | LA | 0.00 | 0.00 | 0.57 | 0.43 | 0.00 | 0.00 | 29.47 | -89.69 |  |
| A03385 | LA | 0.00 | 0.00 | 0.00 | **1.00** | 0.00 | 0.00 | 29.46 | -89.68 | F |
| A03383 | LA | 0.00 | 0.00 | **1.00** | 0.00 | 0.00 | 0.00 | 29.39 | -89.59 | F |
| A03367 | LA | 0.00 | 0.00 | 0.17 | **0.83** | 0.00 | 0.00 | 29.39 | -89.59 | F |
| A03382 | LA | 0.00 | 0.00 | 0.05 | **0.95** | 0.00 | 0.00 | 29.39 | -89.59 | F |
| A03380 | LA | 0.00 | 0.00 | 0.01 | **0.99** | 0.00 | 0.00 | 29.39 | -89.59 | F |
| A03370 | LA | 0.00 | 0.00 | 0.40 | 0.60 | 0.00 | 0.00 | 29.39 | -89.59 |  |
| A03377 | LA | 0.00 | 0.00 | 0.46 | 0.54 | 0.00 | 0.00 | 29.39 | -89.59 |  |
| A03386 | LA | 0.00 | 0.00 | **0.99** | 0.01 | 0.00 | 0.00 | 29.35 | -89.52 | F |
| A03376 | LA | 0.00 | 0.00 | **0.99** | 0.01 | 0.00 | 0.00 | 29.35 | -89.52 | F |
| A03389 | LA | 0.00 | 0.00 | **1.00** | 0.00 | 0.00 | 0.00 | 29.35 | -89.52 | F |
| A03388 | LA | 0.00 | 0.00 | **1.00** | 0.00 | 0.00 | 0.00 | 29.35 | -89.52 | F |
| A03378 | LA | 0.00 | 0.00 | **1.00** | 0.00 | 0.00 | 0.00 | 29.35 | -89.52 | F |
| A03373 | LA | 0.00 | 0.00 | **0.97** | 0.03 | 0.00 | 0.00 | 29.35 | -89.52 | F |
| A03371 | LA | 0.00 | 0.00 | **1.00** | 0.00 | 0.00 | 0.00 | 29.35 | -89.52 | F |
| A03372 | LA | 0.00 | 0.00 | 0.17 | **0.83** | 0.00 | 0.00 | 29.35 | -89.52 | F |
| A03387 | LA | 0.00 | 0.00 | 0.06 | **0.94** | 0.00 | 0.00 | 29.35 | -89.52 | F |
| A03390 | LA | 0.00 | 0.00 | 0.00 | **1.00** | 0.00 | 0.00 | 29.35 | -89.52 | F |
| A03366 | LA | 0.00 | 0.00 | 0.01 | **0.99** | 0.00 | 0.00 | 29.35 | -89.52 | F |
| A03375 | LA | 0.00 | 0.00 | 0.00 | **1.00** | 0.00 | 0.00 | 29.35 | -89.52 | F |
| A03381 | LA | 0.00 | 0.00 | 0.27 | 0.73 | 0.00 | 0.00 | 29.35 | -89.52 |  |
| A03369 | LA | 0.00 | 0.00 | 0.49 | 0.51 | 0.00 | 0.00 | 29.35 | -89.52 |  |
| 12N3830 | MI | 0.00 | 0.00 | **1.00** | 0.00 | 0.00 | 0.00 | 45.95 | -86.02 | C |
| 12N3865 | MI | 0.00 | 0.00 | **0.99** | 0.01 | 0.00 | 0.00 | 45.95 | -86.02 | C |
| 12N3831 | MI | 0.00 | 0.00 | **0.98** | 0.02 | 0.00 | 0.00 | 45.95 | -86.02 | C |
| 12N3833 | MI | 0.00 | 0.00 | **1.00** | 0.00 | 0.00 | 0.00 | 45.95 | -86.02 | C |
| 12N3864 | MI | 0.00 | 0.00 | **0.83** | 0.17 | 0.00 | 0.00 | 45.95 | -86.02 | C |
| 12N3858 | MI | 0.00 | 0.00 | **1.00** | 0.00 | 0.00 | 0.00 | 45.95 | -86.02 | C |
| 12N3843 | MI | 0.00 | 0.00 | **0.88** | 0.12 | 0.00 | 0.00 | 45.95 | -86.02 | C |
| 12N3828 | MI | 0.00 | 0.07 | 0.08 | **0.85** | 0.00 | 0.00 | 45.95 | -86.02 | C |
| 12N3835 | MI | 0.00 | 0.00 | 0.07 | **0.93** | 0.00 | 0.00 | 45.95 | -86.02 | C |
| 12N3851 | MI | 0.00 | 0.00 | 0.01 | **0.99** | 0.00 | 0.00 | 45.95 | -86.02 | C |
| 12N3842 | MI | 0.00 | 0.00 | 0.01 | **0.99** | 0.00 | 0.00 | 45.95 | -86.02 | C |
| 12N3857 | MI | 0.00 | 0.00 | 0.04 | **0.96** | 0.00 | 0.00 | 45.95 | -86.02 | C |
| 12N3856 | MI | 0.00 | 0.00 | 0.01 | **0.99** | 0.00 | 0.00 | 45.95 | -86.02 | C |
| 12N3849 | MI | 0.00 | 0.00 | 0.00 | **1.00** | 0.00 | 0.00 | 45.95 | -86.02 | C |
| 12N3832 | MI | 0.00 | 0.00 | 0.00 | **1.00** | 0.00 | 0.00 | 45.95 | -86.02 | C |
| 12N3848 | MI | 0.00 | 0.00 | 0.00 | **1.00** | 0.00 | 0.00 | 45.95 | -86.02 | C |
| 12N3846 | MI | 0.00 | 0.00 | 0.00 | **1.00** | 0.00 | 0.00 | 45.95 | -86.02 | C |
| 12N3863 | MI | 0.00 | 0.00 | 0.05 | **0.95** | 0.00 | 0.00 | 45.95 | -86.02 | C |
| 12N3860 | MI | 0.00 | 0.00 | 0.80 | 0.20 | 0.00 | 0.00 | 45.95 | -86.02 |  |
| 12N3847 | MI | 0.00 | 0.00 | 0.24 | 0.76 | 0.00 | 0.00 | 45.95 | -86.02 |  |
| 12N3850 | MI | 0.00 | 0.00 | 0.39 | 0.61 | 0.00 | 0.00 | 45.95 | -86.02 |  |
| 12N3855 | MI | 0.00 | 0.00 | 0.69 | 0.31 | 0.00 | 0.00 | 45.95 | -86.02 |  |
| A03127 | FL | 0.00 | 0.00 | **1.00** | 0.00 | 0.00 | 0.00 | 29.78 | -84.86 | G |
| A03275 | FL | 0.00 | 0.00 | 0.17 | **0.83** | 0.00 | 0.00 | 29.66 | -84.85 | G |
| A03277 | FL | 0.00 | 0.00 | 0.28 | 0.72 | 0.00 | 0.00 | 29.66 | -84.85 |  |
| A03185 | FL | 0.00 | 0.00 | 0.66 | 0.34 | 0.00 | 0.00 | 29.66 | -84.85 |  |
| A03181 | FL | 0.00 | 0.00 | **0.96** | 0.04 | 0.00 | 0.00 | 27.89 | -82.84 | G |
| A03132 | FL | 0.00 | 0.00 | **0.98** | 0.02 | 0.00 | 0.00 | 28.90 | -82.61 | G |
| A03129 | FL | 0.00 | 0.00 | 0.36 | 0.64 | 0.00 | 0.00 | 28.90 | -82.61 |  |
| A03272 | FL | 0.00 | 0.00 | **1.00** | 0.00 | 0.00 | 0.00 | 28.89 | -82.59 | G |
| A03187 | FL | 0.00 | 0.00 | 0.00 | **1.00** | 0.00 | 0.00 | 28.89 | -82.59 | G |
| A03278 | FL | 0.00 | 0.00 | 0.00 | **1.00** | 0.00 | 0.00 | 28.89 | -82.59 | G |
| A03282 | FL | 0.00 | 0.00 | 0.56 | 0.44 | 0.00 | 0.00 | 27.95 | -82.45 |  |
| A03273 | FL | 0.00 | 0.00 | 0.00 | **1.00** | 0.00 | 0.00 | 26.44 | -82.11 | G |
| A03188 | FL | 0.00 | 0.00 | 0.09 | **0.91** | 0.00 | 0.00 | 26.44 | -82.10 | G |
| A03274 | FL | 0.00 | 0.00 | 0.00 | **1.00** | 0.00 | 0.00 | 26.44 | -82.10 | G |
| A03276 | FL | 0.00 | 0.00 | 0.00 | **1.00** | 0.00 | 0.00 | 26.44 | -82.10 | G |
| A03184 | FL | 0.00 | 0.00 | 0.59 | 0.41 | 0.00 | 0.00 | 26.44 | -82.10 |  |
| A03130 | FL | 0.00 | 0.00 | **0.98** | 0.02 | 0.00 | 0.00 | 26.44 | -82.07 | G |
| A03128 | FL | 0.00 | 0.00 | **1.00** | 0.00 | 0.00 | 0.00 | 26.44 | -82.07 | G |
| A03131 | FL | 0.00 | 0.00 | **0.89** | 0.11 | 0.00 | 0.00 | 26.44 | -82.07 | G |
| 99N8837 | ON | 0.00 | 0.00 | **1.00** | 0.00 | 0.00 | 0.00 | 43.25 | -81.83 | D |
| 99N8821 | ON | 0.00 | 0.00 | **0.89** | 0.11 | 0.00 | 0.00 | 43.25 | -81.83 | D |
| 99N8818 | ON | 0.00 | 0.00 | **0.95** | 0.05 | 0.00 | 0.00 | 43.25 | -81.83 | D |
| 99N8839 | ON | 0.00 | 0.00 | **0.99** | 0.01 | 0.00 | 0.00 | 43.25 | -81.83 | D |
| 99N8815 | ON | 0.00 | 0.00 | **0.83** | 0.17 | 0.00 | 0.00 | 43.25 | -81.83 | D |
| 99N8823 | ON | 0.00 | 0.00 | 0.00 | **1.00** | 0.00 | 0.00 | 43.25 | -81.83 | D |
| 99N8841 | ON | 0.00 | 0.00 | 0.12 | **0.88** | 0.00 | 0.00 | 43.25 | -81.83 | D |
| 99N8820 | ON | 0.00 | 0.00 | 0.00 | **1.00** | 0.00 | 0.00 | 43.25 | -81.83 | D |
| 99N8832 | ON | 0.00 | 0.00 | 0.03 | **0.97** | 0.00 | 0.00 | 43.25 | -81.83 | D |
| 99N8829 | ON | 0.00 | 0.01 | 0.15 | **0.85** | 0.00 | 0.00 | 43.25 | -81.83 | D |
| 99N8831 | ON | 0.00 | 0.00 | 0.06 | **0.94** | 0.00 | 0.00 | 43.25 | -81.83 | D |
| 99N8816 | ON | 0.00 | 0.00 | 0.01 | **0.99** | 0.00 | 0.00 | 43.25 | -81.83 | D |
| 99N8828 | ON | 0.00 | 0.00 | 0.09 | **0.91** | 0.00 | 0.00 | 43.25 | -81.83 | D |
| 99N8842 | ON | 0.00 | 0.00 | 0.00 | **1.00** | 0.00 | 0.00 | 43.25 | -81.83 | D |
| 99N8826 | ON | 0.00 | 0.00 | 0.01 | **0.99** | 0.00 | 0.00 | 43.25 | -81.83 | D |
| 99N8825 | ON | 0.00 | 0.00 | 0.00 | **1.00** | 0.00 | 0.00 | 43.25 | -81.83 | D |
| 99N8808 | ON | 0.00 | 0.00 | 0.00 | **1.00** | 0.00 | 0.00 | 43.25 | -81.83 | D |
| 99N8806 | ON | 0.00 | 0.00 | 0.00 | **1.00** | 0.00 | 0.00 | 43.25 | -81.83 | D |
| 99N8812 | ON | 0.00 | 0.00 | 0.00 | **1.00** | 0.00 | 0.00 | 43.25 | -81.83 | D |
| 99N8811 | ON | 0.00 | 0.00 | 0.00 | **1.00** | 0.00 | 0.00 | 43.25 | -81.83 | D |
| 99N8840 | ON | 0.00 | 0.00 | 0.76 | 0.24 | 0.00 | 0.00 | 43.25 | -81.83 |  |
| 99N8822 | ON | 0.00 | 0.00 | 0.78 | 0.22 | 0.00 | 0.00 | 43.25 | -81.83 |  |
| 99N8810 | ON | 0.00 | 0.00 | 0.77 | 0.23 | 0.00 | 0.00 | 43.25 | -81.83 |  |
| A03280 | FL | 0.00 | 0.00 | **1.00** | 0.00 | 0.00 | 0.00 | 24.66 | -81.35 | G |
| A03133 | FL | 0.00 | 0.00 | 0.00 | **1.00** | 0.00 | 0.00 | 24.71 | -81.06 | G |
| A03134 | FL | 0.00 | 0.00 | 0.00 | **1.00** | 0.00 | 0.00 | 24.71 | -81.06 | G |
| A03177 | FL | 0.00 | 0.00 | 0.00 | **1.00** | 0.00 | 0.00 | 25.12 | -80.40 | G |
| A03201 | FL | 0.00 | 0.00 | 0.00 | **0.98** | 0.00 | 0.02 | 26.19 | -80.37 | G |
| A03283 | FL | 0.00 | 0.00 | 0.79 | 0.21 | 0.00 | 0.00 | 26.19 | -80.37 |  |
| A03180 | FL | 0.00 | 0.00 | 0.00 | **1.00** | 0.00 | 0.00 | 26.01 | -80.15 | G |
| A03281 | FL | 0.00 | 0.00 | 0.00 | **1.00** | 0.00 | 0.00 | 25.78 | -80.13 | G |
| A03279 | FL | 0.00 | 0.00 | 0.00 | **1.00** | 0.00 | 0.00 | 25.98 | -80.12 | G |
| A03178 | FL | 0.00 | 0.00 | **1.00** | 0.00 | 0.00 | 0.00 | 26.07 | -80.11 | G |
| A03179 | FL | 0.00 | 0.00 | 0.04 | **0.96** | 0.00 | 0.00 | 26.23 | -80.08 | G |
| A03342 | MD | 0.00 | 0.00 | 0.00 | 0.00 | 0.00 | **1.00** | 38.29 | -75.12 | E |
| A03343 | DE | 0.00 | 0.00 | 0.00 | 0.07 | 0.00 | **0.93** | 38.53 | -75.05 | E |
| A03322 | CT | 0.00 | 0.00 | 0.00 | **1.00** | 0.00 | 0.00 | 41.28 | -72.67 | E |
| A03337 | RI | 0.00 | 0.00 | 0.08 | **0.92** | 0.00 | 0.00 | 41.30 | -71.87 | E |
| A03339 | RI | 0.00 | 0.00 | 0.06 | **0.94** | 0.00 | 0.00 | 41.34 | -71.70 | E |
| A03311 | RI | 0.00 | 0.00 | 0.02 | **0.98** | 0.00 | 0.00 | 41.16 | -71.57 | E |
| A03309 | RI | 0.00 | 0.00 | 0.00 | **1.00** | 0.00 | 0.00 | 41.59 | -71.44 | E |
| A03315 | MA | 0.00 | 0.00 | 0.00 | **0.88** | 0.00 | 0.12 | 41.76 | -70.71 | E |
| A03361 | MA | 0.00 | 0.00 | 0.00 | 0.08 | 0.00 | **0.92** | 41.55 | -70.53 | E |
| A03364 | MA | 0.00 | 0.00 | 0.00 | **0.92** | 0.00 | 0.08 | 41.79 | -70.52 | E |
| A03338 | MA | 0.00 | 0.00 | 0.03 | **0.92** | 0.00 | 0.05 | 41.37 | -70.47 | E |
| A03314 | MA | 0.00 | 0.00 | 0.00 | **1.00** | 0.00 | 0.00 | 41.56 | -70.46 | E |
| A03356 | MA | 0.00 | 0.00 | 0.00 | **1.00** | 0.00 | 0.00 | 41.64 | -70.24 | E |
| A03359 | ME | 0.00 | 0.00 | 0.00 | **1.00** | 0.00 | 0.00 | 43.57 | -70.21 | E |
| A03360 | MA | 0.00 | 0.00 | 0.05 | **0.95** | 0.00 | 0.00 | 41.64 | -70.18 | E |
| A03313 | MA | 0.00 | 0.00 | 0.00 | **1.00** | 0.00 | 0.00 | 42.08 | -70.18 | E |
| A03328 | MA | 0.00 | 0.00 | 0.00 | **0.93** | 0.00 | 0.07 | 41.28 | -70.11 | E |
| A03331 | MA | 0.00 | 0.00 | 0.00 | 0.02 | 0.00 | **0.98** | 41.28 | -70.11 | E |
| A03329 | ME | 0.00 | 0.00 | 0.00 | **0.99** | 0.00 | 0.01 | 43.81 | -70.10 | E |
| A03345 | MA | 0.00 | 0.00 | 0.00 | 0.00 | 0.00 | **1.00** | 41.66 | -70.09 | E |
| A03354 | MA | 0.00 | 0.00 | 0.78 | 0.22 | 0.00 | 0.00 | 41.90 | -70.02 |  |
| A03332 | ME | 0.00 | 0.00 | 0.72 | 0.28 | 0.00 | 0.00 | 44.13 | -68.87 |  |
| A03316 | ME | 0.00 | 0.00 | 0.00 | 0.00 | 0.00 | **1.00** | 44.37 | -68.26 | E |
| A03327 | ME | 0.00 | 0.00 | 0.00 | 0.00 | 0.00 | **1.00** | 44.91 | -67.07 | E |

Table S4. Rubias self-assignments of individuals of known origins to their respective conservation units. Percentages are based on the number of samples out of the total samples from each conservation unit that were assigned with > 80% confidence to that (bold) or another conservation unit. The conservation units were delineated as described in the text. We tested assignment accuracy using additional samples genotyped by the Fluidigm method that were not used for delineation of the conservation units, and because we lacked additional for eastern Canada we also tested assignments using combined Fluidigm and RADseq genotyped samples. The top half of the table shows assignment results using only the additional Fluidigm genotyped samples and the bottom half shows the assignment results using both the RADseq and Fluidigm genotyped samples.

|  | **Conservation unit** | **Alaska** | **Pacific Northwest** | **central Canada** | **Midwest** | **eastern Canada** | **New England** | **Number sampled** |
| --- | --- | --- | --- | --- | --- | --- | --- | --- |
| **Fluidigm Only** | **Alaska** | **0.96** | 0.00 | 0.00 | 0.04 | NA | 0.00 | 23 |
|  | **Pacific Northwest** | 0.00 | **0.94** | 0.00 | 0.00 | NA | 0.00 | 18 |
|  | **central Canada** | 0.00 | 0.00 | **0.82** | 0.18 | NA | 0.00 | 11 |
|  | **Midwest** | 0.00 | 0.02 | 0.00 | **0.92** | NA | 0.03 | 64 |
|  | **eastern Canada** | NA | NA | NA | NA | **NA** | NA | 0 |
|  | **New England** | 0.00 | 0.00 | 0.00 | 0.00 | NA | **0.98** | 57 |
| **RADseq Included** | **Alaska** | **0.92** | 0.00 | 0.00 | 0.04 | 0.00 | 0.00 | 24 |
|  | **Pacific Northwest** | 0.00 | **0.90** | 0.08 | 0.03 | 0.00 | 0.00 | 40 |
|  | **central Canada** | 0.00 | 0.00 | **0.84** | 0.07 | 0.02 | 0.00 | 43 |
|  | **Midwest** | 0.00 | 0.03 | 0.03 | **0.89** | 0.00 | 0.03 | 72 |
|  | **eastern Canada** | 0.00 | 0.00 | 0.00 | 0.00 | **1.00** | 0.00 | 11 |
|  | **New England** | 0.00 | 0.00 | 0.00 | 0.00 | 0.00 | **0.97** | 110 |

Table S5. *F*_ST_ values and confidence intervals between 16 sampling regions (primarily defined as US states and Canadian provinces), based on the RADseq PE dataset.

|  |  | **Pacific Northwest** | | **North Central** | | | | | **Midwest** | | **Canada_East** | | **New England** | | | | |
| --- | --- | --- | --- | --- | --- | --- | --- | --- | --- | --- | --- | --- | --- | --- | --- | --- | --- |
| **Interior West** | **Reg** | **MT** | **BC** | **MAN** | **SK** | **WY** | **AB** | **ONT_W** | **MI3** | **WI2** | **ONT_E** | **QB** | **MA** | **ME** | **NB** | **NH** | **NY** |
|  | **MT** |  | 0.014 | 0.041 | 0.025 | 0.020 | 0.022 | 0.034 | 0.058 | 0.059 | 0.035 | 0.047 | 0.079 | 0.064 | 0.067 | 0.069 | 0.057 |
| **North Central** | **BC** | 0.013 - 0.015 |  | 0.047 | 0.030 | 0.023 | 0.024 | 0.039 | 0.064 | 0.068 | 0.041 | 0.053 | 0.084 | 0.070 | 0.074 | 0.075 | 0.063 |
|  | **MAN** | 0.040 - 0.043 | 0.045 - 0.048 |  | 0.016 | 0.017 | 0.017 | 0.018 | 0.041 | 0.044 | 0.018 | 0.027 | 0.060 | 0.046 | 0.051 | 0.050 | 0.037 |
|  | **SK** | 0.024 - 0.026 | 0.029 - 0.031 | 0.015 - 0.017 |  | 0.007 | 0.003 | 0.011 | 0.034 | 0.035 | 0.009 | 0.023 | 0.055 | 0.041 | 0.043 | 0.046 | 0.033 |
|  | **WY** | 0.019 - 0.021 | 0.021 - 0.024 | 0.016 - 0.019 | 0.006 - 0.008 |  | 0.005 | 0.010 | 0.035 | 0.036 | 0.010 | 0.022 | 0.055 | 0.041 | 0.042 | 0.045 | 0.032 |
|  | **AB** | 0.021 - 0.023 | 0.023 - 0.025 | 0.016 - 0.019 | 0.002 - 0.004 | 0.004 - 0.006 |  | 0.010 | 0.033 | 0.032 | 0.007 | 0.021 | 0.055 | 0.042 | 0.044 | 0.046 | 0.031 |
|  | **ONT_W** | 0.033 - 0.036 | 0.037 - 0.041 | 0.016 - 0.020 | 0.010 - 0.012 | 0.009 - 0.012 | 0.008 - 0.012 |  | 0.024 | 0.027 | 0.001 | 0.009 | 0.042 | 0.028 | 0.029 | 0.033 | 0.017 |
| **Midwest** | **MI3** | 0.056 - 0.059 | 0.062 - 0.065 | 0.039 - 0.042 | 0.032 - 0.035 | 0.034 - 0.036 | 0.032 - 0.035 | 0.023 - 0.026 |  | 0.038 | 0.023 | 0.026 | 0.055 | 0.041 | 0.045 | 0.045 | 0.033 |
|  | **WI2** | 0.057 - 0.061 | 0.065 - 0.070 | 0.042 - 0.046 | 0.033 - 0.036 | 0.034 - 0.037 | 0.030 - 0.034 | 0.025 - 0.029 | 0.036 - 0.039 |  | 0.026 | 0.028 | 0.062 | 0.046 | 0.053 | 0.051 | 0.037 |
| **Canada East** | **ONT_E** | 0.034 - 0.037 | 0.039 - 0.044 | 0.016 - 0.020 | 0.008 - 0.010 | 0.008 - 0.011 | 0.006 - 0.009 | 0 - 0.003 | 0.0212 - 0.024 | 0.023 - 0.028 |  | 0.005 | 0.037 | 0.023 | 0.026 | 0.029 | 0.012 |
|  | **QB** | 0.045 - 0.049 | 0.051 - 0.055 | 0.025 - 0.029 | 0.022 - 0.024 | 0.020 0.023 | 0.019 - 0.022 | 0.007 - 0.010 | 0.025 - 0.028 | 0.026 - 0.030 | 0.003 - 0.007 |  | 0.034 | 0.019 | 0.023 | 0.025 | 0.011 |
| **New England** | **MA** | 0.077 - 0.080 | 0.082 - 0.086 | 0.059 - 0.062 | 0.054 - 0.057 | 0.054 - 0.057 | 0.053 - 0.056 | 0.041 - 0.044 | 0.053 - 0.056 | 0.060 - 0.064 | 0.036 - 0.039 | 0.033 - 0.035 |  | 0.015 | 0.025 | 0.005 | 0.021 |
|  | **ME** | 0.063 - 0.066 | 0.068 - 0.072 | 0.044 - 0.048 | 0.041 - 0.043 | 0.040 - 0.042 | 0.040 - 0.043 | 0.027 - 0.029 | 0.040 - 0.042 | 0.044 - 0.047 | 0.022 - 0.025 | 0.018 - 0.021 | 0.014 - 0.015 |  | 0.007 | 0.004 | 0.008 |
|  | **NB** | 0.066 - 0.069 | 0.072 - 0.076 | 0.049 - 0.053 | 0.042 - 0.045 | 0.040 - 0.044 | 0.042 - 0.046 | 0.028 - 0.031 | 0.043 - 0.046 | 0.050 - 0.055 | 0.024 - 0.028 | 0.021 - 0.025 | 0.023 - 0.026 | 0.006 - 0.008 |  | 0.013 | 0.011 |
|  | **NH** | 0.0667 - 0.070 | 0.073 - 0.076 | 0.048 - 0.052 | 0.045 - 0.047 | 0.044 - 0.046 | 0.045 - 0.047 | 0.032 - 0.035 | 0.044 - 0.046 | 0.049 - 0.053 | 0.027 - 0.030 | 0.023 - 0.026 | 0.004 - 0.006 | 0.003 - 0.004 | 0.012 - 0.014 |  | 0.012 |
|  | **NY** | 0.055 - 0.059 | 0.061 - 0.065 | 0.035 - 0.039 | 0.032 - 0.034 | 0.031 - 0.034 | 0.030 0.033 | 0.016 - 0.019 | 0.032 - 0.034 | 0.035 - 0.039 | 0.010 - 0.014 | 0.010 - 0.013 | 0.020 - 0.022 | 0.007 - 0.008 | 0.009 - 0.012 | 0.011 - 0.013 |  |

Figure S1. PCA of genetic differentiation among common loon breeding populations in North America. Analysis is based on 106 individuals and 1,852 bi-allelic polymorphisms from the ddRAD-seq dataset. PC1, which accounts for 3.95% of the genetic variation, captures geographic differentiation roughly along a west to east axis, whereas PC2 (2.39% of variation) further separates the Alaska population and, to a lesser extent, populations in New England and the Maritimes.


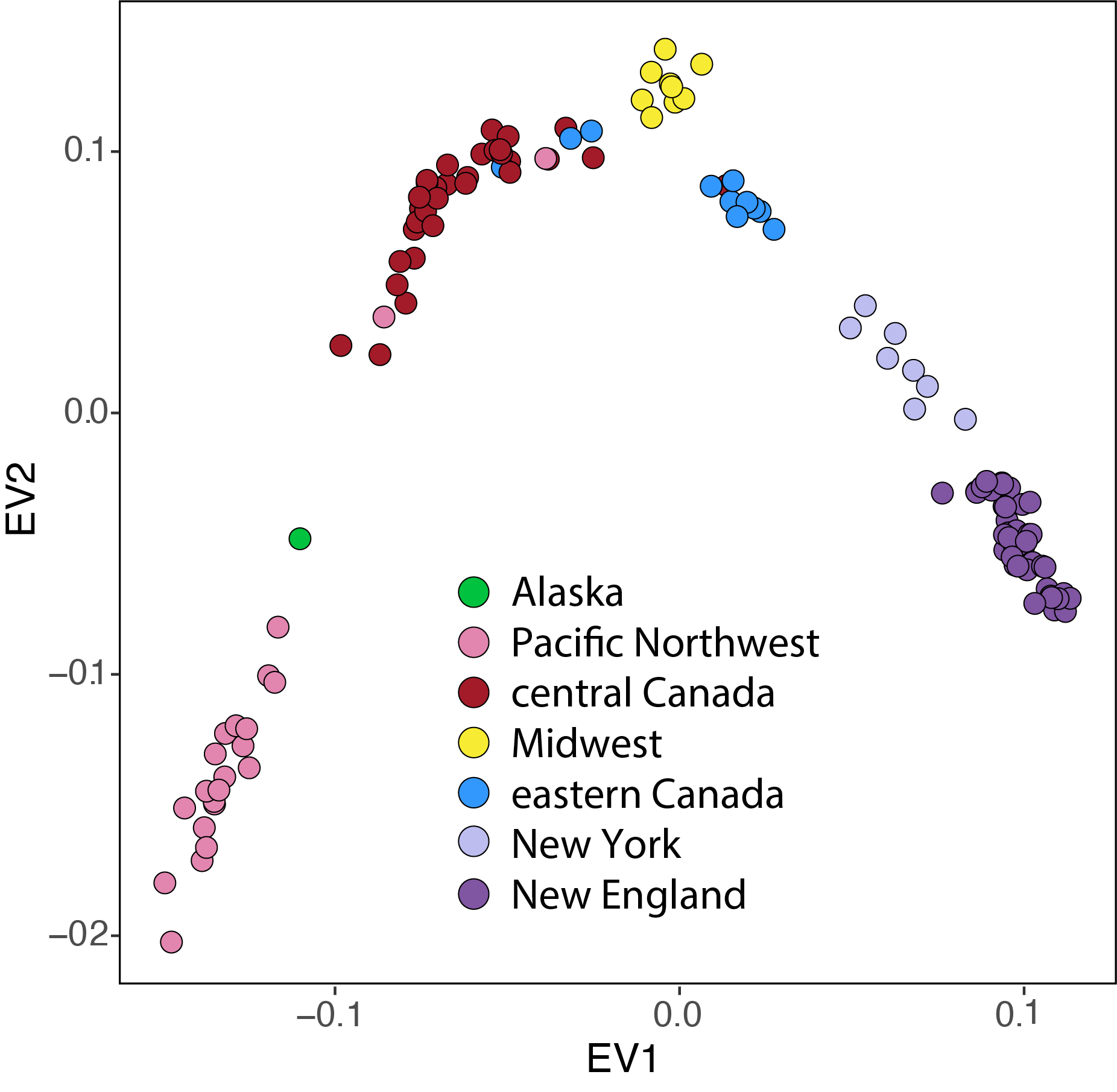


Figure S2. Principal Components Analysis of population structure using RAD-PE sequencing data (129 samples, 39,912 SNPs). New York clusters separately here, but in later analyses is indistinguishable from the New England cluster.


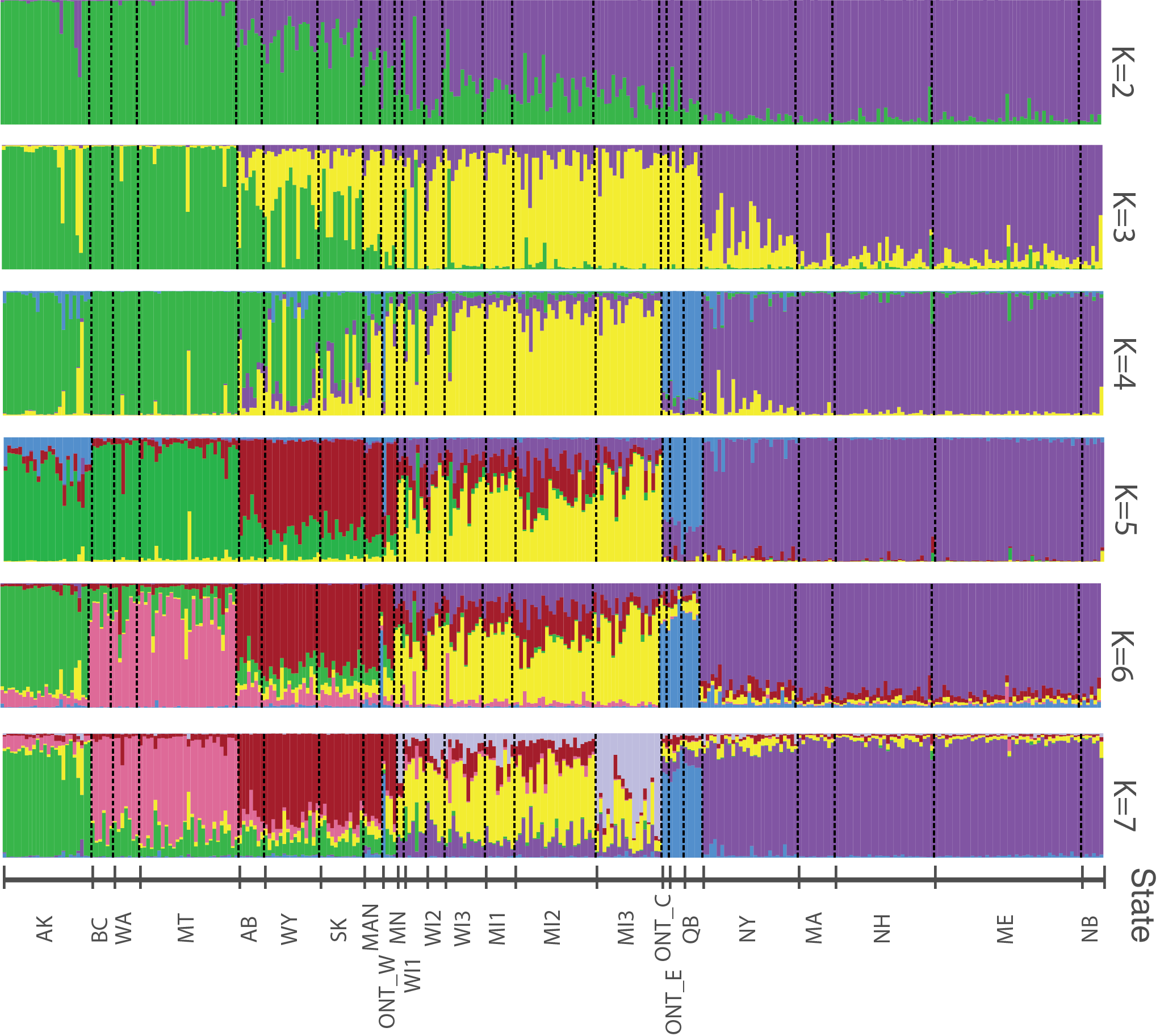


Figure S3. *STRUCTURE* analysis of population structure using the Fluidigm SNP dataset.


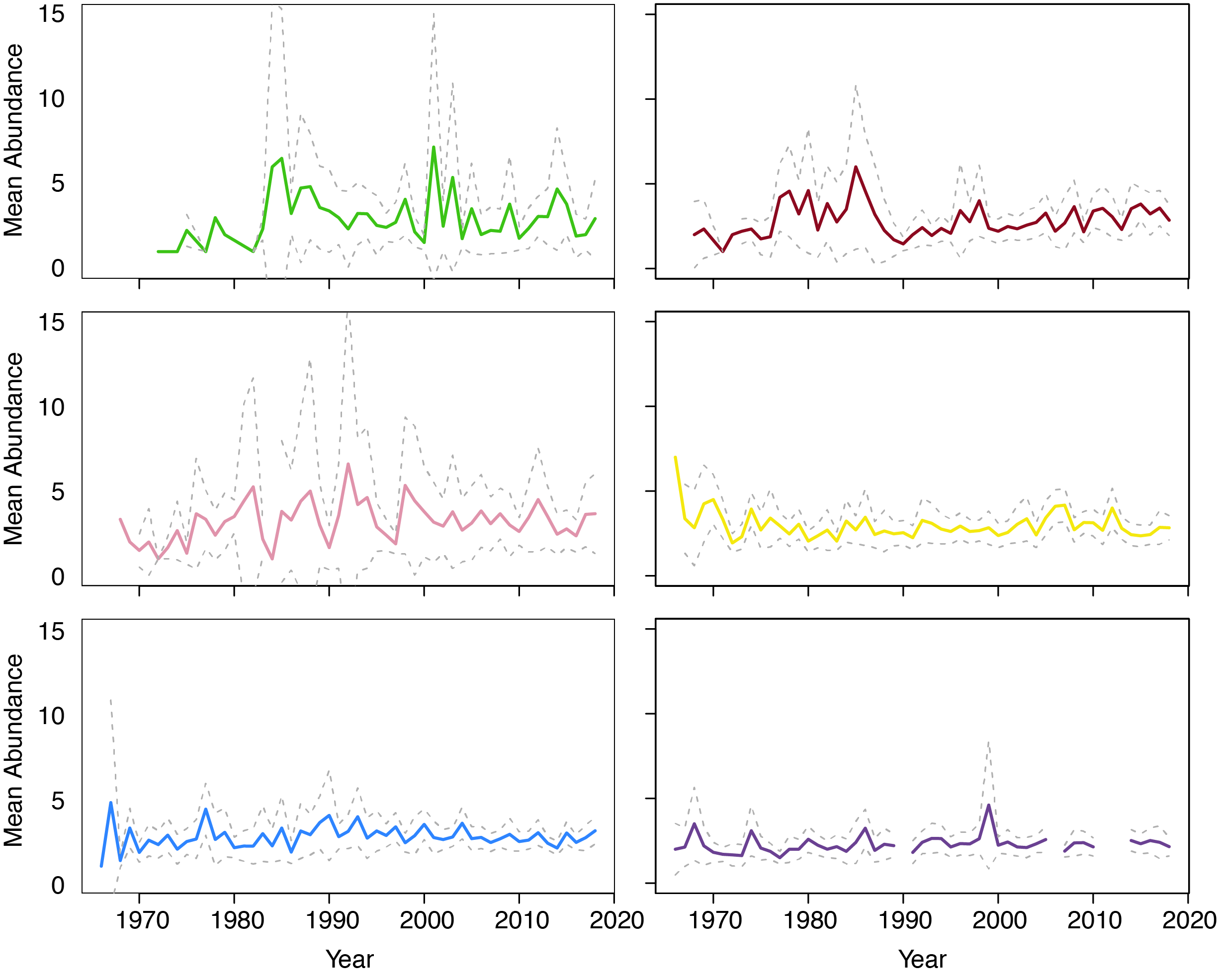


Figure S4. Common loon population trends from BBS data. Mean abundance is the average abundance within each unit of data collected along standardized routes (24.5 miles long with 3-minute point counts conducted at intervals of 0.5 miles along the length of the route). Pacific Northwest (orange) trend = 0.003, F = 0049_1,363_, *P* = 0.82; central Canada (red) trend = 0.010, F = 1.32_1,796_, *P* = 0.25; New England (purple) trend = 0.004, F = 0.58_1,1163_, *P* = 0.44; eastern Canada (blue) trend = -0.001, F = 0.067_1,1338_, *P* = 0.80; Midwest (yellow) trend = 0.003, F = 0.28_1,1335_, *P* = 0.60; Alaska (green) trend = -0.009, F = 0.23_1,418_, *P* = 0.63.


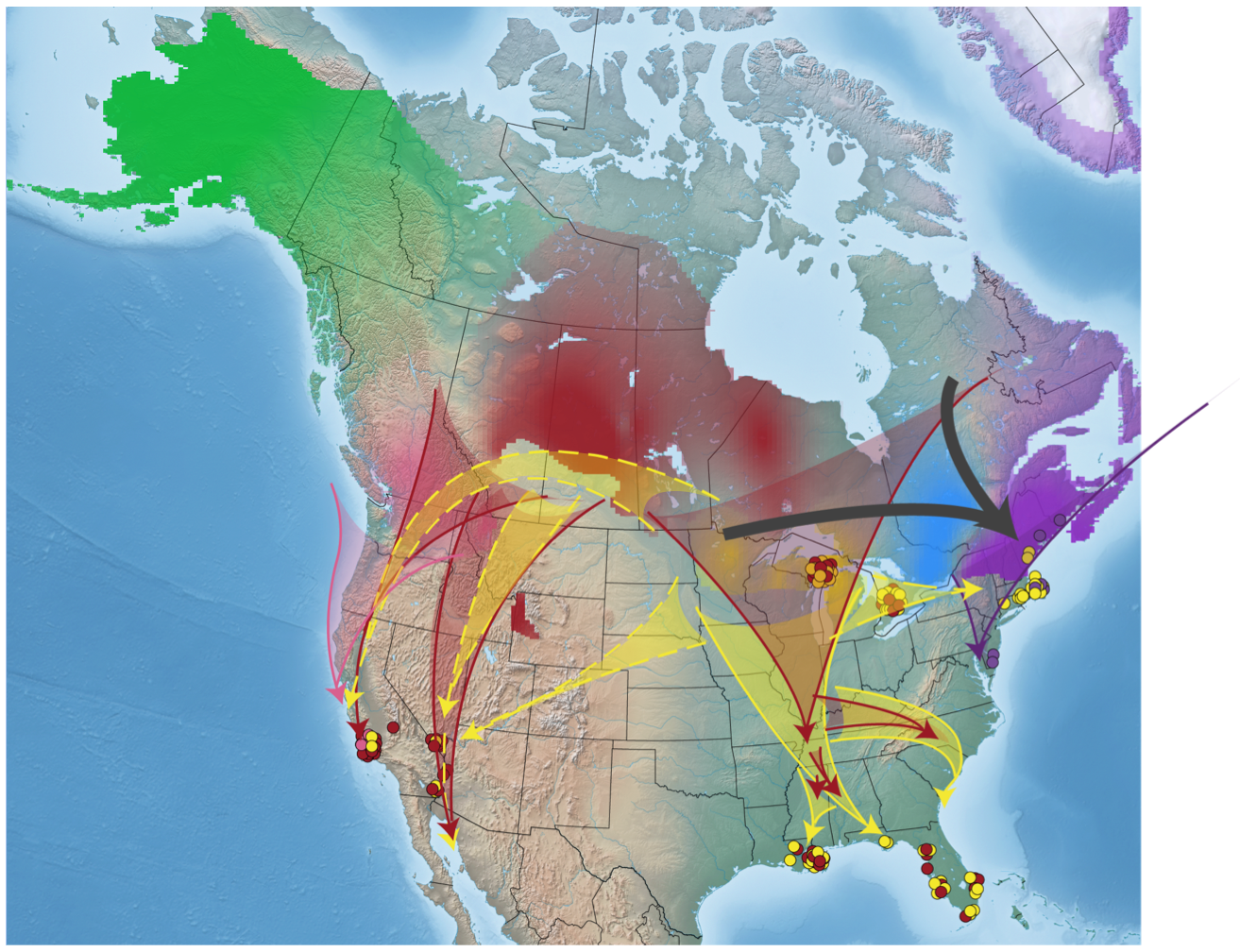


Figure S5. Potential migratory pathways for common loons as suggested by the identified linkages between breeding, migratory and wintering areas. Pathways are colored according to conservation unit: Pacific Northwest (orange), central Canada (red), Midwest (yellow) and New England (purple). We are unable to suggest pathways for Alaska (green) or eastern Canada (blue) due to a lack of non-breeding samples linked to those two units. Pathways outlined by solid lines are those for which our data and Birds of North America (BNA) data are in agreement. Those with dashed outlines are ones for which a pathway has not previously been suggested. The thick gray arrows are pathways suggested by BNA data that aren’t supported by our data.


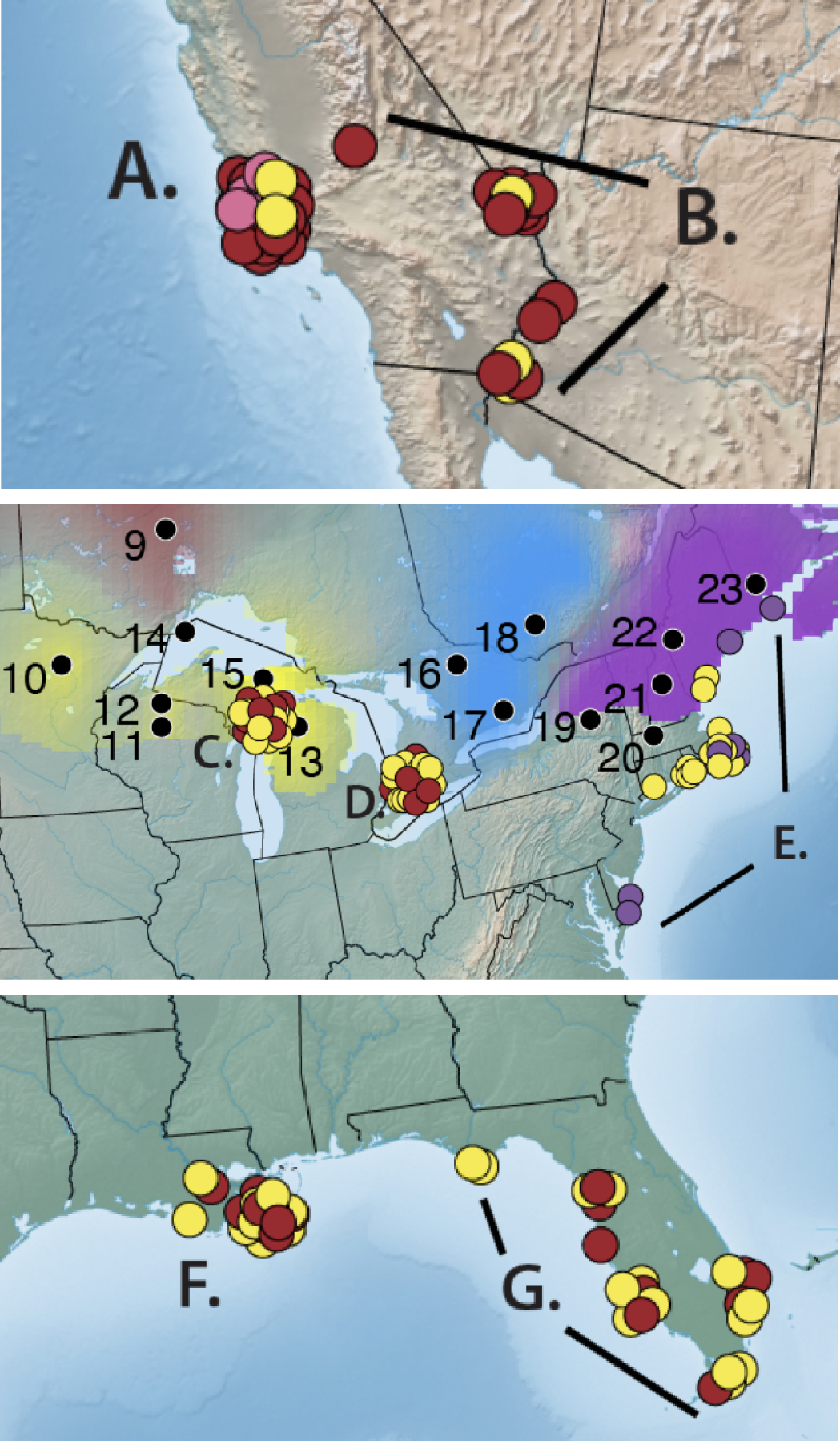


Figure S6. Close-ups of assignment maps from main text Figure 1. Note that A, C, D and the large cluster in F represent multiple samples taken at a single location.
